# TT-Nb_2_O_5_ with dual-band modulation and exceptional performance retention for fast-responsive electrochromics

**DOI:** 10.1093/nsr/nwaf154

**Published:** 2025-04-24

**Authors:** Qingjiao Huang, Peipei Shao, Mingao Hou, Yihan Lei, Zhexuan Ou, Jiacheng Hu, Ying Zhu, Bowen Li, Menghan Yin, Yiwen Zhang, Renfu Zhang, Jiawei Sun, Changjian Li, Guangfu Luo, Rui-Tao Wen

**Affiliations:** Department of Materials Science and Engineering, Southern University of Science and Technology, Shenzhen 518055, China; Department of Materials Science and Engineering, Southern University of Science and Technology, Shenzhen 518055, China; Department of Materials Science and Engineering, Southern University of Science and Technology, Shenzhen 518055, China; Department of Materials Science and Engineering, Southern University of Science and Technology, Shenzhen 518055, China; Department of Materials Science and Engineering, Southern University of Science and Technology, Shenzhen 518055, China; Department of Materials Science and Engineering, Southern University of Science and Technology, Shenzhen 518055, China; Department of Materials Science and Engineering, Southern University of Science and Technology, Shenzhen 518055, China; Department of Materials Science and Engineering, Southern University of Science and Technology, Shenzhen 518055, China; Department of Materials Science and Engineering, Southern University of Science and Technology, Shenzhen 518055, China; Department of Materials Science and Engineering, Southern University of Science and Technology, Shenzhen 518055, China; Department of Materials Science and Engineering, Southern University of Science and Technology, Shenzhen 518055, China; Department of Materials Science and Engineering, Southern University of Science and Technology, Shenzhen 518055, China; Department of Materials Science and Engineering, Southern University of Science and Technology, Shenzhen 518055, China; Guangdong Provincial Key Laboratory of Functional Oxide Materials and Devices, Southern University of Science and Technology, Shenzhen 518055, China; Department of Materials Science and Engineering, Southern University of Science and Technology, Shenzhen 518055, China; Institute of Innovative Materials, Southern University of Science and Technology, Shenzhen 518055, China; Department of Materials Science and Engineering, Southern University of Science and Technology, Shenzhen 518055, China; Guangdong Provincial Key Laboratory of Functional Oxide Materials and Devices, Southern University of Science and Technology, Shenzhen 518055, China

**Keywords:** TT-Nb_2_O_5_, dual-band modulation, performance retention, fast switching kinetics

## Abstract

Independently modulating the transmittance of solar spectra, specifically within the visible and near-infrared light ranges, presents a significant prospect for windows that can effectively manage lighting and energy consumption in both buildings and electrical-vehicles. Electrochromic devices, capable of regulating the transmittance of visible and near-infrared light in response to external electrical stimuli, are considered as one of the ideal candidates for smart windows. However, electrochromic devices typically suffer from single-mode control (i.e. simultaneously varying the visible and near-infrared light), slow response and inadequate long-term durability. In this paper, we demonstrate that TT-Nb₂O₅ enables independent modulation of visible and near-infrared light and possesses rapid switching kinetics and exceptional cycling stability, i.e. no observed degradation of optical modulation after more than 10 000 cycles. The dual band modulation is attributed to a combination of progressive splitting and downward shift of Nb 3*d* conduction bands and rise of Fermi level as ion insertion proceeds. The open framework of the crystal structures accounts for the exceptional cycling stability. Simulation results based on assembled smart windows indicate a potential cooling energy saving of 160 GJ without compromising the outdoor view, or 225 GJ for a complete blocking of visible and near-infrared light can be achieved in hot climate zones.

## Teaser

This work reports a novel electrochromic oxide that achieves independent modulation of visible and near-infrared light, alongside fast switching dynamics and excellent long-term stability.

## INTRODUCTION

Dual-band modulation refers to the independent control of the visible (VIS) and near-infrared (NIR) segments of the solar spectrum, empowering electrochromic materials or devices to independently regulate both VIS and NIR [1–6]. Electrochromic materials embodying dual-band modulation, fast switching and long-term cycling lifetime are the ‘holy grail’ for reducing energy consumption of VIS and NIR in buildings and vehicles. So far, amorphous oxides remain the top contenders for advancing high-performance electrochromic devices. This preference is attributed to the solid solution occurring upon ion intercalation, a process deemed comparatively fast when contrasted with the phase transition in crystalline. Amorphous WO_3_ (denoted as *a*-WO_3_) is the most studied and used electrochromic material, which possesses larger optical modulation (especially the VIS range) and faster switching than the crystalline ones (i.e. monoclinic WO_3_, denoted as *c*-WO_3_). However, *a*-WO_3_ suffers from poor cycling lifetime due to the ion trapping effect [7–10], leading to a degraded colored state upon cycling. It should be pointed out that ion trapping induced degradation is ubiquitous in amorphous cathodic electrochromic oxides, at least in sputtered ones [7,11–13]. To achieve dual-band modulation, surface localized plasma resonance (LSPR) has been recently combined into electrochromic materials to independently modulate the NIR part, in which nanocrystals (NCs) have to be employed. Milliron *et al.* employed a composite approach and demonstrated that the dispersed ITO NCs in an amorphous NbO*_x_* matrix can achieve dual-band modulation [4]. The NIR modulation was induced by LSPR absorption around the ITO NCs through pairing electrons upon ion insertion into amorphous NbO*_x_*, while VIS modulation was achieved *via* polaron hopping in the NbO*_x_* glassy matrix. Given the time-consuming and costly nature of the synthesis process for ITO-NbO_x_ composite films, a single-component of electrochromic material is more desirable. Following this concept, electrochromic NCs with high electron density at the surface could also achieve LSPR induced NIR absorption under pairing electron insertion. Subsequently, monoclinic WO_3-_*_x_* and anatase TiO_2-_*_x_* NCs showed rapid modulation of NIR light at the initial stage of capacitive charge insertion [14,15]. However, VIS modulation is relatively slow, since further electron/ion insertions led to phase transitions in NCs from monoclinic WO_3-_*_x_* to tetragonal/cubic WO_3-_*_x_*, and anatase TiO_2-_*_x_* to orthorhombic TiO_2-_*_x_*, [14–16]. Meanwhile, the *Δ*T (denoted as transmittance at the bleached state (*T*_b_) minus transmittance at the colored state (*T*_c_) at a given wavelength) was also found to degrade rapidly; for instance, WO_3-_*_x_* NCs lost 8.3% optical modulation after only 1000 cycles, very likely due to the volume change induced mechanical failure upon phase transitions [14]. It is important to note that LSPR requires dispersed NCs which is challenging for non-liquid electrolyte based full devices, especially considering the main stream of massive magnetron sputtering for electrochromic materials deposition. To date, no satisfactory solution has been found to integrate both dual-band modulation and rapid kinetics within a structurally stable framework.

In this paper, we report that TT-Nb_2_O_5_ (pseudo-hexagonal) thin films possess dual-band modulation with rapid-switching kinetics and exceptional performance retention. As ion insertion proceeds, Nb 3*d* conduction bands split off into sub-bands, which gradually move toward the valence bands, while the Fermi level rises steadily due to band filling. The dual-band modulation of TT-Nb_2_O_5_ is thus realized through a combined effect of sub-conduction band downward shift and increase of Fermi level, which is distinctly different from the commonly employed NCs for LSPR induced NIR modulation. Thus, dual-band modulation is no longer restricted to the NCs format, making it feasible for massive and direct multilayer deposition. We find that the two-dimensional (2D) layered structure of TT-Nb_2_O_5_ possess a ‘room and pillar’ framework with very low steric hindrance for fast Li-ion diffusion, superior than the prevailing *a*-WO_3_, T-Nb_2_O_5_ (orthorhombic), and other reported cathodic electrochromic oxides [17–20]. The negligible volume variation and absence of phase transition leads to no observable degradation of electrochromic performance after more than 10^4^ cycles. Dual-band modulation can be maintained in a full device by employing TT-Nb_2_O_5_ and non-electrochromic active tungsten vanadium oxide (NbVO_5_). Simulations demonstrate that, in a typical medium-sized office building equipped with smart windows possessing features of dual-band modulations, the potential cooling energy savings in hot climate zones can reach up to 160 GJ without affecting the outdoor view, or 225 GJ for a complete blocking of both VIS and NIR, which is superior to the traditional WO_3_/NiO_x_ configuration. This energy reduction is equivalent to a substantial annual reduction of 31.5 and 44.5 metric tons in CO_2_ emissions.

## RESULTS AND DISCUSSION

### Dual-band modulation of TT-Nb_2_O_5_

**Figure 1.**
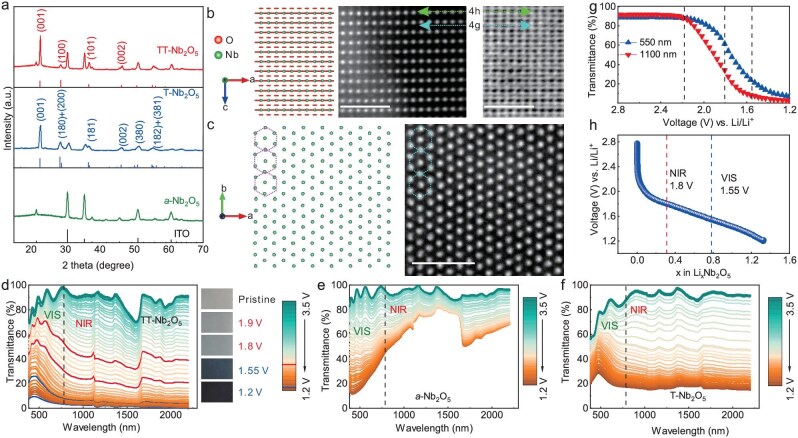
Structure and optical variation of Nb_2_O_5_ thin films. (a) The XRD patterns of Nb_2_O_5_ thin films with different polymorphs (*a*-Nb_2_O_5_, TT-Nb_2_O_5_ and T-Nb_2_O_5_). Indium tin oxide (ITO) is listed as a reference (JCPDS:71–2194, black vertical line). TT-Nb_2_O_5_ (JCPDS: 28–0317, red line) and T-Nb_2_O_5_ (JCPDS: 30–0873, blue line) were obtained by annealing of *a*-Nb_2_O_5_ thin films at 600°C and 850°C for 2 hours. The thin films were deposited on ITO/quartz glass and subjected to post annealing treatment in air atmosphere. The TT-Nb_2_O_5_ thin films deposited on both ITO/glass and ITO/quartz are haze-free and possess identical electrochromic properties (as shown in Fig. S2). (b and c) Cross-sectional HAADF- and ADF-STEM images of a TT-Nb_2_O_5_ thin-film viewing along *b*-axis and *c*-axis, respectively. The 4h and 4g layers are marked which are constructed by NbO_2_ and O. The red and green spheres denote as O and Nb atoms. Purple and blue dotted hexagons are intended to illustrate a one-to-one correspondence between theoretical and experimental results. The scale bar is 2 nm. (d–f) The *in situ* optical transmittance variation of TT-, *a*- and T-Nb_2_O_5_ thin films, respectively. The thickness of the films was 330 nm for all phases. The sweep rate was 1 mV s^−1^, in a three-electrode configuration. The red bold curve represents the *cold* mode (1.90 V and 1.80 V), while the blue bold curve represents the dark mode (1.55 V and 1.20 V), along with the corresponding digital photographs. The vertical dash line was drawn to separate the VIS and NIR. The ‘*dip*’ ∼1600 nm is due to strong absorption from the electrolyte. (g) Single wavelength of 550 and 1100 nm of TT-Nb_2_O_5_ was recorded in real time as potential decreasing in a CV cycle. (h) The concentration of inserted Li, *x*, as a function of applied potential in TT-Nb_2_O_5_ at a sweep rate of 1 mV s^−1^.

As both amorphous (*a*-) and T-Nb_2_O_5_ are electrochromically active [21,22], we compare TT-Nb_2_O_5_ with them in parallel. The as-deposited Nb_2_O_5_ is amorphous, and TT- and T-Nb_2_O_5_ phases were obtained through post-annealing, as shown in Fig. 1a and Fig. S1. All the Nb_2_O_5_ thin films were transparent at the pristine state. We used scanning transmission electron microscopy (STEM) to investigate the atomic configuration of the TT-Nb_2_O_5_. High angle annular dark field (HAADF) and annular dark field (ADF) images of TT-Nb_2_O_5_, viewed from the *b*-axis, are shown in Fig 1b. The 4h and 4g layers are marked, and it can be observed that Nb atoms locate at 4 h, and the 4 g layer is only composed by oxygen atoms. HAADF image viewed from the *c*- axis is presented in Fig. 1c, which clearly shows the hexagonal pattern, as expected.

The potential was linearly swept from 3.5 V (open circuit potential, OCP) to 1.2 V vs. Li (the potential refers to Li/Li^+^ unless otherwise specified) while recording the variation of spectra in real time. As shown in Fig. 1d–f, when compared to T- and *a*-Nb_2_O_5_, TT-Nb_2_O_5_ exhibits exceptional optical modulation in the VIS and NIR range (350–2500 nm), i.e. *ΔT*>80%. At the colored state (i.e. 1.2 V), the full spectra approach zero, suggesting a complete blocking of VIS and NIR. Moreover, TT-Nb_2_O_5_ possesses distinct dual-band modulation. It can be found that prior to the potential reaching 1.8 V, the optical modulation is mainly from NIR (Fig. 1d, 1.8 V in red, where the color of the film rarely varies as shown in the digital image in the inset). As the potential further decreases from 1.8 V to 1.2 V, both the VIS and NIR drop (Fig. 1d, 1.2 V in blue where the film totally changes to dark blue). This selective modulation of VIS and NIR offers the feasibility to achieve three working modes for electrochromic devices: *bright* mode (both VIS and NIR are highly transparent), *cold* mode (transparent for VIS but not for NIR), and *dark* mode (both VIS and NIR are blocked). T-Nb_2_O_5_ also demonstrates the feature of dual-band modulation, which, however, is less profound than TT-Nb_2_O_5_; in contrast, the optical modulation of *a*-Nb_2_O_5_ is predominantly limited in the VIS range (Fig. 1e and f, Fig. S3). An intuitive picture of dual-band modulation of TT-Nb_2_O_5_ is provided in Fig. 1g, showing that NIR (1100 nm) starts to drop earlier than VIS (550 nm). In order to quantitatively analyze the dual-band modulation for TT-Nb_2_O_5_ as a function of the inserted Li quantity, the incorporated *x* value in Li*_x_*Nb_2_O_5_ of TT-phase was calculated by $x = \frac{{{\mathrm{QM}}}}{{{\mathrm{eA\rho d}}{N}_A}}$ through the redox process:


(1) \begin{eqnarray*}
{\mathrm{N}}{{\mathrm{b}}}_2{{\mathrm{O}}}_5 + x{\mathrm{L}}{{\mathrm{i}}}^ + + x{{\mathrm{e}}}^ - \leftrightarrow {\mathrm{L}}{{\mathrm{i}}}_x{\mathrm{N}}{{\mathrm{b}}}_2{{\mathrm{O}}}_5.
\end{eqnarray*}

Here, Q is the inserted charge that can be derived from CV data, M is molar mass, and e is the elementary charge. A, ρ and d are intercalated area, density and thickness of the film. *N_A_* is Avogadro's constant. It can be observed in Fig. 1h that the modulation primarily occurs in the NIR light when *x* <0.3, beyond which both VIS and NIR are modulated.

**Figure 2.**
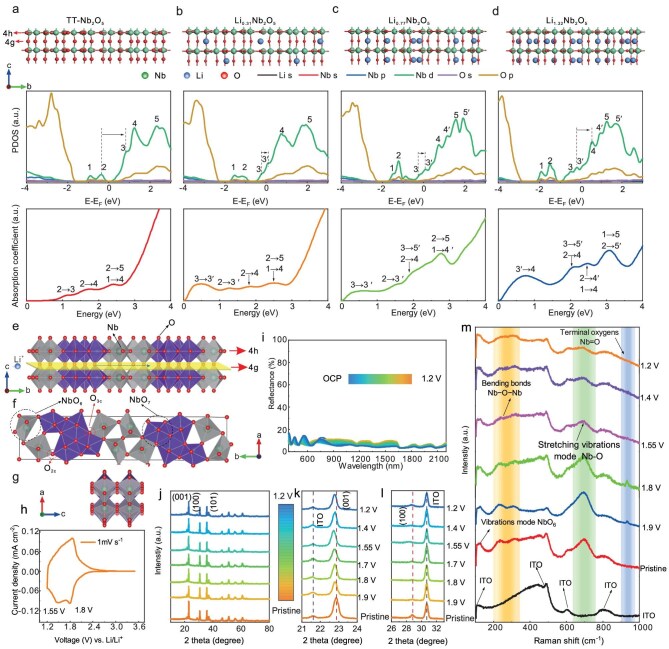
Electrochromic process of TT-Nb_2_O_5_. (a–d) Geometrical structure, projected density of states, and optical absorption spectra for TT-Nb_2_O_5_, Li_0.31_Nb_2_O_5_, Li_0.77_Nb_2_O_5_, and Li_1.32_Nb_2_O_5_, respectively. The structural configuration of TT-Nb_2_O_5_ is composed of alternating NbO_2_ layers (4h) and oxygen layers (4g). The electron transitions contributing to each optical absorption peak are indicated by black arrows. (e–g) The 1 × 1 × 2 superstructure of TT-Nb_2_O_5_ viewing along *a*-axis, *c*-axis and b-axis, respectively. The schematic illustration of the Li ion diffusion layer (4g) is marked in yellow. Distorted octahedra (NbO_6_) and pentagonal bipyramidal (NbO_7_) are also revealed in grey and purple. The O_2c_ and O_3c_ refer to two distinct oxygen coordination’s in the 4h layer, which are active sites for Li location. (h) The CV curve of TT-Nb_2_O_5_ with a sweep rate of 1 mV s^−1^ in the range of 1.2–3.5 V vs. Li. The reversible potential window has been explored and confirmed to be 1.2–3.5 V, as shown in Fig. S7. (i) *In situ* reflectance spectra of TT-Nb_2_O_5_ thin film as potential decreases. The results showed that the reflectance rarely varied during the entire intercalation process (i.e. <10%), confirming that the optical transmittance variation is from absorption, rather than reflection. (j) *Ex situ* XRD patterns of TT-Nb_2_O_5_ film at marked potentials, representing different Li concentrations. (k–l) Enlarged view of (001) and (100) planes of TT-Nb_2_O_5_. The unchanged position of the ITO peak is marked as a reference. (m) Raman spectra of TT-Nb_2_O_5_ at different potentials.

To uncover the origin of the dual-band modulation, we perform first-principles calculations based on density functional theory (DFT) to investigate the geometrical, electronic, and optical properties of TT-Li*_x_*Nb_2_O_5_ (*x* = 0, 0.31, 0.77, and 1.32). The optimized lattice constants are fully consistent with the experimental results as shown in Fig. 1a. Here, the PBE + U method is employed, the accuracy of which is comparable to the more advanced HSE06 function (Fig. S4). The optimized structures and projected density of states changes with the concentration of Li ions in TT-Nb_2_O_5_ are illustrated in Fig. 2a–d (see Materials and Methods for more details of the optimized structures of all the calculated systems). Electronic structure calculations reveal that the valence bands of Li*_x_*Nb_2_O_5_ are primarily composed of O 2*p* orbitals, while the conduction bands are predominantly contributed by Nb 3*d* orbitals. As shown in Fig. 2a–d, the Nb 3*d* conduction bands split into sub-bands and shift progressively toward the valence bands upon Li ion insertion, likely due to the strain effects induced by Li intercalation. Concurrently, the Fermi level rises due to electron doping by the intercalated Li ions. These two key features lead to significant changes in the optical absorption spectra. In the pure TT-Nb_2_O_5_ phase (Fig. 2a), the lowest electron transitions occur between states 2 and 3 (2→3), with no obvious adsorption features in the NIR region. This is consistent with the high transmittance observed experimentally in the NIR region (Fig. 1d).

Figure 2e–g illustrates a supercell of 1 × 1 × 2 construction with a polyhedral configuration by Nb and O atoms in three axes. It can be seen that the pseudohexagonal structure is mainly composed of highly distorted octahedral (NbO_6_) and pentagonal bipyramidal (NbO_7_) [17,23,24], forming an alternating pattern of loosely packed (4g) and densely packed (4h) layers with a two-dimensional layered structure characteristic. The 4h layer is a hybrid Nb-O layer and each Nb atom is coordinated by 4 or 5 oxygen atoms in the *ab*-plane; the bridge oxygens in Nb-O-Nb, i.e. the oxygen in the 4g layer, links two 4h layers. As observed from the *b*-axis (Fig. 2g), there is an empty channel configured by distorted octahedral (NbO_6_) and pentagonal bipyramidal (NbO_7_) for Li diffusion in the 4g layer. Here, three categories of oxygen coordination environments are identified (Fig. S5): O_3c_ and O_2c_ which bond to three and two Nb atoms in the 4h layer are active sites for Li location [24,25], and O_4g_ locates in the 4g oxygen plane, as marked in Fig. 2e. At the stage of TT-Li_0.31_Nb_2_O_5_, DFT calculations reveal that the inserted Li ions locate at the 4g layer and bond to two O_3c_, as shown in Fig. S5. The splitting of state 3 leads to an additional transition of 3→3’, creating a new absorption peak ∼0.50 eV. This results in the decrease of optical transmittance in the NIR region (see Fig. 2b and Fig. 1d).

With a further increase of lithium content to 0.77, a new transition of 3→5’ gives rise to an absorption peak ∼2.0 eV (Fig. 2c), which significantly reduces the optical transmittance in the VIS region, as observed experimentally (Fig. 1d). At this moment, in addition to Li-O_3c_ bonding, the inserted Li atoms in the 4g layer start to bond to two O_2c_ (Fig. S5). At a higher Li content of 1.32, the 3→3’ transition disappears. Simultaneously, a new transition of 3’→4 emerges, which contributes to an increased NIR absorption due to the large density of state 4. Additionally, new transitions of 2→4’and 2→5’ further enhance the optical absorption in the VIS region. In the cyclic voltammetry (CV) curve, an oxidation peak was first confirmed at 1.8 V (*x* = 0.31, displayed in Fig. 2h), followed by another oxidation at 1.55 V, which is fully consistent with the DFT predication for the two Li intercalation sites, i.e. one between two O_3c_ and the other between two O_2c_. X-ray photoelectron spectroscopy (XPS) analysis revealed that Nb^5+^ was partially reduced to Nb^4+^ at *x* = 0.31 and both Nb^4+^ and Nb^3+^ were present at *x* = 0.77 (Fig. S6). In summary, Li ion insertion induces the splitting and downward shift of the unoccupied Nb 3*d* bands, as well as the upward shift of the Fermi level. These changes result in an initial increase in the NIR absorption at lower Li concentration, followed by enhanced optical absorption in both the visible and NIR regions as the Li content further increases. DFT calculations show that the entire ion insertion is a solid-solution process without phase transition. We found that the change in reflectance at different potentials is negligible (Fig. 2i), affirming that the variation in optical spectra is due to absorption upon ion insertion, rather than metallic *Drude* reflection [26], consistent with the DFT calculations.

It has been reported that small polaron hopping of W^6+^↔W^5+^ in amorphous electrochromic WO_3_ resulted in an absorption peak centered at ∼800 nm (also see Fig. S8), leading to a color change from transparent to dark blue [27]. Further ion/electron pair insertion can generate W^4+^ and yield optical absorption at the VIS through bipolaron hopping of W^6+^↔W^4+^ [28]. However, the formed W^4+^ is irreversible and bipolaron hopping of W^6+^↔W^4+^ suppresses the small polaron hopping of W^6+^↔W^5+^, rendering the dual band modulation of WO_3_ impossible [29]. Moreover, the initial optical absorption centers at 0.5 eV (∼2500 nm) as Li is inserted, which is far more red-shifted than amorphous WO_3_, making the dual band modulation in TT-Nb_2_O_5_ more substantial.

XRD results (Fig. 2j–l) of TT-Nb_2_O_5_ showed that under various potentials (*x* up to 1.32), the pseudohexagonal structure is maintained, suggesting Li diffusion in TT-Nb_2_O_5_ is in a manner of solid solution rather than phase changes, which is consistent with our DFT calculations. This is also different from T-Nb_2_O_5_ where phase transition from orthorhombic to monoclinic takes place as *x* reaches 0.8 (Fig. S9). The slight shift of (001) peak to lower angles of TT-Nb_2_O_5_ suggests that the lattice constant along *c*-axis is expanded upon ion insertion. The weak diffraction intensity of (100) peak becomes more diffuse as potential drops, suggesting a disordered arrangement in the *ab* plane. Raman spectra (Fig. 2m) also confirmed that intercalation of Li ions in the 4g layers bond to O_3c_ sites of NbO_6_ (4h layer) at the initial stage of ion insertion. This process was accompanied by the concurrent development of Nb^4+^, as evidenced by the emergence of a distinct vibration mode associated with terminal Nb=O bonds at 932 cm^−1^ [30,31]. Peaks centered at ∼108 cm^−1^, 488 cm^−1^, 602 cm^−1^and ∼800 cm^−1^ are assigned to ITO/glass [32,33]. Raman peaks in the range of 624–770 cm^−1^, originally from the stretching vibrations of Nb-O bonds in 4h layers [24], were unvaried at this stage, suggesting the configurations of 4h layers were rarely affected and supported the conclusion that the inserted Li ions locate at 4g layers. As the potential further drops from 1.80 V to 1.20 V, the Raman peaks from vibration of octahedra (NbO_6_, at ∼122 cm^−1^), bending bonds of Nb-O-Nb (∼228 and 308 cm^−1^) and Nb-O bonds (∼695 cm^−1^) in 4h layers [24,31,34] gradually decline and disappear, providing compelling evidence for the Nb-O bonds breaking in 4h layers. During the Li ion intercalation process, DFT calculations show that the maximum volume expansion was 4.3%. As the TT-Nb_2_O_5_ is cycled with a sweep rate of 20 mV s^−1^, the maximum volume change is only 0.5% (Fig. S10), when the *ab* plane is considered to be unvariable due to the very low diffraction intensity for (100) peak. Such a small volume change suggests an intrinsic high cycling stability of TT-Nb_2_O_5_ as will be discussed layer.

### Fast kinetics and excellent performance retention

**Figure 3.**
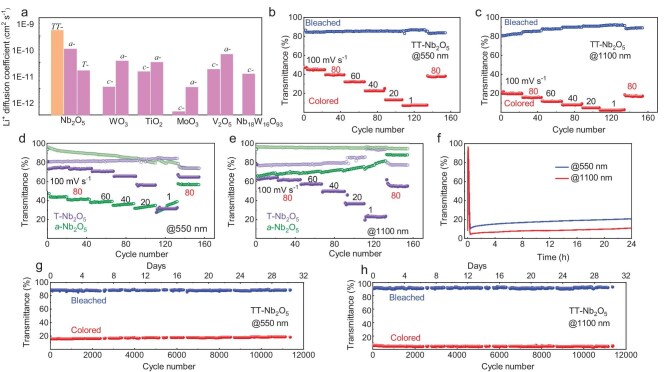
Fast kinetics in TT-Nb_2_O_5_. (a) The comparison of diffusion coefficients between different polymorphs of Nb_2_O_5_ and other cathode electrochromic oxides; *a-* and *c*- denote as amorphous and crystalline. The crystalline metal oxide thin films are WO_3_ (monoclinic), MoO_3_ (monoclinic), V_2_O_5_ (orthorhombic), TiO_2_ (tetragonal) and Nb_18_W_16_O_93_ (orthorhombic). (b and c) ***Δ***T as a function of various sweep rates for 550 nm (VIS) and 1100 nm (NIR) in TT-Nb_2_O_5_, respectively. The corresponding sweep rate was marked. (d and e) ***Δ***T as a function of sweep rate for VIS (550 nm) and NIR (1100 nm) in T-Nb_2_O_5_ and *a*-Nb_2_O_5_. (f) The optical memory effect of TT-Nb_2_O_5_ thin films. The potential was cut after the bias reached 1.2 V. (g and h) VIS and NIR performance retention of TT-Nb_2_O_5_ thin films. The measurements were conducted in the range 1.2–3.5 V vs. Li/Li^+^ at a sweep rate of 20 mV s^−1^.

Nb_2_O_5_, in the forms of amorphous (*a*-), pseudo hexagonal (TT-) and orthorhombic (T-), showed intercalation pseudo-capacitance [35], suggesting that ion storage predominantly takes place within the bulk rather than on the surface (Fig. S11, XPS of depth profile in Fig. S12, Supplementary note 1 and also XRD and Raman as shown later). For a quantitative and comprehensive comparison, the lithium diffusion coefficients of the well-studied cathodic electrochromic oxides, in the forms of both amorphous and crystalline phases, were calculated through the following equation [36,37]:


(2) \begin{eqnarray*}
{i}_p = \left( {2.69 \times {{10}}^5} \right){n}^{\frac{3}{2}}{\mathrm{A}}{D}^{\frac{1}{2}}{v}^{\frac{1}{2}}{C}_0,
\end{eqnarray*}

where *D* is the diffusion coefficient in cm^2^ s^−1^, *i_p_* is the anodic peak current of the CV curve, *n* is the number of electrons (which is 1 in current case), *C_0_* is the concentration of ions in the electrolyte (1 mol L^−1^); $v\ $is the sweep rate (20 mV s^−1^). A is the effective area of the sample immersed in the solution. As shown in Fig. 3a, the diffusion coefficient of TT-Nb_2_O_5_ is 1.65 × 10^−9^ cm^2^ s^−1^ at room temperature and is significantly higher than the ones for *a*- and T-Nb_2_O_5_, as well as all other cathodic electrochromic oxide thin films. We obtained all the metal oxide thin films through magnetron sputtering with optimized parameters (see Methods and Figs S13–S17), and their Li diffusion coefficients were comparable with those reported [18,20,38,39]. Additionally, it can be noted that all amorphous phases have a larger Li diffusion coefficient than the crystalline ones, where TT-Nb_2_O_5_ is the only exception. Meanwhile, the galvanostatic intermittent titration technique (GITT) and electrochemical impedance spectroscopy (EIS) were further carried out to verify the electrochemical kinetic characteristics of different polymorphs of Nb_2_O_5_ and other cathode electrochromic oxides (Figs S18–S22, Supplementary note 2). Consistently, TT-Nb_2_O_5_ exhibited superior electrochemical kinetic properties compared to other cathode electrochromic oxides.

Figure 3b and c plot the real-time *Δ*T of VIS (550 nm) and NIR (1100 nm) of TT-Nb_2_O_5_ as a function of sweep rate in the potential range of 1.2–3.5 V. It shows that TT-Nb_2_O_5_ possesses excellent reversibility and can maintain unprecedented *Δ*T throughout the entire sweep rate from 100 to 1 mV s^−1^, which indicates there is an extremely fast kinetics in terms of ion transportation. Especially, a negligible difference of the *Δ*T can be observed when the rate is increased by a factor of 20 from 1 to 20 mV s^−1^. As a comparison in Fig. 3d and e, *a*-Nb_2_O_5_ also possesses fast kinetics, however, severe ion trapping upon cycling (Fig. S10) leads to decreased *T*_b_ and *Δ*T [29]. Although T-Nb_2_O_5_ has shown dual-band modulation, the enlarged difference of *Δ*T as the sweep rate decreases implies a slow ion diffusion, a more distinct difference can be found when comparing the *Δ*T between 1 and 20 mV s^−1^. Overall, the rate performance of T-Nb_2_O_5_ is obviously lower than that of TT-Nb_2_O_5_. Here, we also want to note that although the charge capacity of TT-Nb_2_O_5_ is quite different between 1 and 20 mV s^−1^ (Fig. S11), there is negligible difference in *Δ*T, suggesting not all the inserted charge contributes to coloration. In other words, for an electrochromic electrode, once the optical transmittance decreases to zero at the colored state (*Δ*T is maximum at this point), further charge insertion is no longer needed, even though the host matrix may still have room to accommodate more ions. In this context, the prevention of mechanical instability or degradation induced by potential structure rearrangement upon a large number of ion incorporation can be effectively ensured.

The unprecedented *Δ*T from both VIS and NIR at high sweep rate of TT-Nb_2_O_5_ signifies that its crystal structure allows exceptionally fast ionic transport. As observed from the *b*-axis in Fig. 2g, there is an empty channel along the *b*-axis in the 4g layer that allows fast ion transportation. This is different from T-Nb_2_O_5_ in that although T-Nb_2_O_5_ has a similar configuration as TT-Nb_2_O_5_, inserted Li ions also accommodate in 4g layers [25]. In T-Nb_2_O_5_, there is an arrangement of 0.8 Nb atoms in the 4g layer in a unit cell (Fig. S23), which ‘decelerates’ Li diffusion as proved from the measured diffusion coefficient in Fig. 3a.

The negligible volume change (0.5%) also suggests excellent cycling stability. Indeed, as shown in Fig. 3g–h, there is unprecedented *Δ*T and non-observed degradation of both VIS and NIR after more than 10^4^ cycles (equivalent to 31 days) for TT-Nb_2_O_5_, which benefits from the open 4g layer with very low steric hindrance (Fig. 2g). It should be pointed out that T-Nb_2_O_5_ also possesses excellent cycling stability because of the open 4g layer, i.e. no degradation after 10^4^ cycles (Fig. S10), however, as discussed above, the 0.8 Nb atom in 4g layer ‘decelerates’ ion diffusion. Therefore, at a high sweep rate, there is only ∼40% of the *Δ*T due to relatively low kinetics compared to >80% of *Δ*T in TT-Nb_2_O_5_. In contrast to TT- and T-, *a*-Nb_2_O_5_ exhibits poor performance retention as indicated by the obvious degradation within 100 cycles (Fig. 3d and e).

In addition to the dual-band modulation, rapid-switching and excellent performance retention, the ‘optical memory effect’ is another important figure-of-merit for electrochromic materials, representing the persistence of a *mode* without power supply. The pristine state of TT-Nb_2_O_5_ is transparent and naturally stable. When the potential was withdrawn after reaching a colored state (Fig. 3f), we found that the VIS and NIR only increased by 10% and 7% after 24 hours, confirming an excellent optical memory effect and its potential use for very low power consumption devices.

### Smart windows with dual-band modulation for global energy saving and CO_2_ emission reduction

**Figure 4.**
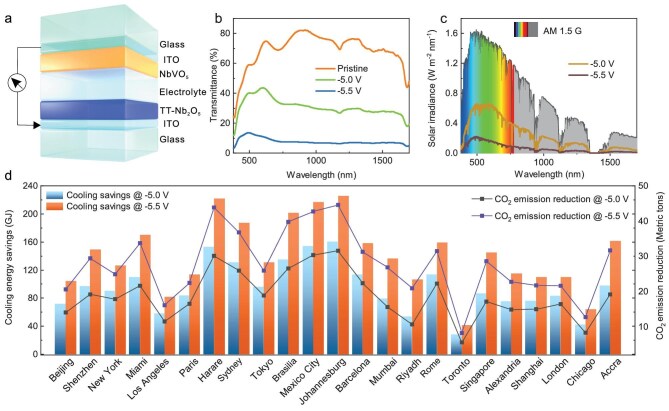
Dynamic modulation and evaluation of energy savings all over the world. (a) The structure diagram of a solid-state electrochromic device with dual-band modulations. (b) Transmittance spectra of a full electrochromic device based on TT-Nb_2_O_5_ and NbVO_5_ electrodes at various voltages. (c) Solar irradiance spectra converted from (b). (d) Predicted global cooling energy saving and CO_2_ emission reduction per capita.

The full devices by employing TT-Nb_2_O_5_ and NbVO_5_ electrodes (Fig. 4a) exhibited dual-band modulation characteristics. To avoid interference of the dual band modulation from the ion storage layer, an electrochromic inactive layer (Fig. S24) of niobium vanadium oxide (NbVO_5_) was used to well match the TT-Nb_2_O_5_ thin film. The associated assembly process of electrochromic devices is provided in Materials and Methods of the supporting information. We operated the devices through chronoamperometry (CA) within the potential range from open circuit potential of 0.17 V to −5.5 V, and recorded the variation of spectra in real-time. As shown in Fig. 4b, the electrochromic devices exhibit exceptional dual-band modulation: prior to the voltage reaching −5.0 V, the main optical modulation is from NIR; as the voltage further decreases from −5.0 V to −5.5 V, the VIS and NIR also decrease. The optical modulation in the VIS and NIR ranges is 50% (550 nm) and 70% (1100 nm), respectively. The transmittance spectra of the electrochromic devices were converted to solar irradiance spectra in the wavelength region of 380–1700 nm (Fig. 4c), which demonstrated excellent performance for solar irradiation modulation. In the *cool* mode (−5.0 V), the electrochromic devices block solar heat in the NIR region while maintaining a high VIS transmittance for daylighting. Therefore, the *cool* mode can significantly reduce energy consumption for air conditioning and lighting. In the *dark* mode (−5.5 V), the electrochromic devices almost totally block out solar radiation. We also compare with WO_3_/NiO_x_-based smart windows (Fig. S25); the consistent optical evolution observed under electrical stimulation indicates a deficiency in its dual-band modulation capabilities.

To further investigate the thermal management ability and applicability of TT-Nb_2_O_5_ thin film in practice at both modes, we conducted a building energy consumption simulation in a typical full-scale medium-size office building model to predict the annual cooling energy saving performance for all over the world. The medium-size office building prototype (DOE pre-1980) developed by the U.S. Department of Energy has 3 floors with an aspect ratio of 1.5 (length, 49.91 m; width, 33.27 m; height, 10.66 m) and glazing fraction of 0.33 (more information can be found in Supplementary note 3) [40]. Based on this building prototype, the simulation program established a baseline energy consumption pattern and only modified the components as well as optical properties of exterior windows on the basis of TT-Nb_2_O_5_/NbVO_5_ smart windows at various states, i.e. *cool* mode and *dark* mode, to predict a cooling energy saving pattern as shown in Fig. 4d.

Here, we selected representative cities in different climate zones and more typical hot locations (shown in Fig. 4d) based on the Koppen-Geiger classification to extend the results to the whole world in order to demonstrate the potential of cooling energy saving [41]. For all locations, our electrochromic smart windows exhibited effective annual cooling energy savings and CO_2_ emission reduction [42,43] at dual modes, as shown in Fig. 4d (more information can be found in Supplementary note 3). Specifically, the cities, such as Johannesburg in the Republic of South Africa (160.37 GJ at *cool* mode, 225.83 GJ at *dark* mode), Shenzhen in China (97.35 GJ, 148.82 GJ), Brasilia in Brazil (134.96 GJ, 201.4 GJ), Sydney in Australia (131.3 GJ, 187.1 GJ), Miami in America (109.91 GJ, 170.27 GJ), and Singapore (86.82 GJ, 145.03 GJ) in particular have exceptional potential energy savings at both modes to release the energy pinch, especially for cities with higher urban density. It is worth to emphasize that during periods with excessive exposed sunlight in these locations in Fig. 4d, the *cool* mode can render efficient NIR blocking without losing visual contact with the outdoors, making substantial cooling energy savings possible.

## CONCLUSION

In summary, we report that TT-Nb_2_O_5_ thin films possess dual-band modulation, fast switching kinetics and exceptional performance retention. Specifically, the dual-band modulation of TT-Nb_2_O_5_ is induced by a combined effect of Fermi level ascension and Nb 3*d* conduction band splitting and downward shift. When *x* = 0.3, the inserted Li ions coordinate with O_3c_, which induces near-infrared absorption. When *x* further increases to 0.77, the continuously inserted Li ions bond to O_2c_, thereby triggering more extensive splitting of Nb 3*d* orbitals and culminating in the absorption of visible light. The TT-Nb_2_O_5_ has a distinct two-dimensional (2D) layer structure arranged with alternating dense and loose atomic layers. The loosely packed 4g layers facilitate fast Li migration with minimal steric hindrance, resulting in the largest lithium diffusion coefficient in TT-Nb_2_O_5_ when compared to the rest of reported cathodic electrochromic oxides. No degradation was observed after over 10^4^ cycles due to its negligible volume expansion and no phase transition. Furthermore, we achieved full electrochromic smart windows possessing dual-band modulation and found that when the full smart windows are employed in a typical medium-size office building, the HVAC system can save up to 160 GJ without affecting outdoor view, or 225 GJ for a complete VIS and NIR blocking. We believe that electrochromic devices exhibiting rapid switching, dual band modulation and high stability upon ion intercalation can promote the development of electrochromic community and other technologies.

## Supplementary Material

nwaf154_Supplemental_File
