# Correction to: TT-Nb_2_O_5_ with dual-band modulation and exceptional performance retention for fast-responsive electrochromics

**DOI:** 10.1093/nsr/nwag061

**Published:** 2026-02-20

**Authors:** Qingjiao Huang, Peipei Shao, Mingao Hou, Yihan Lei, Zhexuan Ou, Jiacheng Hu, Ying Zhu, Bowen Li, Menghan Yin, Yiwen Zhang, Renfu Zhang, Jiawei Sun, Changjian Li, Guangfu Luo, Rui-Tao Wen

**Affiliations:** Department of Materials Science and Engineering, Southern University of Science and Technology, Shenzhen 518055, China; Department of Materials Science and Engineering, Southern University of Science and Technology, Shenzhen 518055, China; Department of Materials Science and Engineering, Southern University of Science and Technology, Shenzhen 518055, China; Department of Materials Science and Engineering, Southern University of Science and Technology, Shenzhen 518055, China; Department of Materials Science and Engineering, Southern University of Science and Technology, Shenzhen 518055, China; Department of Materials Science and Engineering, Southern University of Science and Technology, Shenzhen 518055, China; Department of Materials Science and Engineering, Southern University of Science and Technology, Shenzhen 518055, China; Department of Materials Science and Engineering, Southern University of Science and Technology, Shenzhen 518055, China; Department of Materials Science and Engineering, Southern University of Science and Technology, Shenzhen 518055, China; Department of Materials Science and Engineering, Southern University of Science and Technology, Shenzhen 518055, China; Department of Materials Science and Engineering, Southern University of Science and Technology, Shenzhen 518055, China; Department of Materials Science and Engineering, Southern University of Science and Technology, Shenzhen 518055, China; Department of Materials Science and Engineering, Southern University of Science and Technology, Shenzhen 518055, China; Guangdong Provincial Key Laboratory of Functional Oxide Materials and Devices, Southern University of Science and Technology, Shenzhen 518055, China; Department of Materials Science and Engineering, Southern University of Science and Technology, Shenzhen 518055, China; Institute of Innovative Materials, Southern University of Science and Technology, Shenzhen 518055, China; Department of Materials Science and Engineering, Southern University of Science and Technology, Shenzhen 518055, China; Guangdong Provincial Key Laboratory of Functional Oxide Materials and Devices, Southern University of Science and Technology, Shenzhen 518055, China

In the article by Huang *et al*., ‘TT-Nb_2_O_5_ with dual-band modulation and exceptional performance retention for fast-responsive electrochromics’ (*National Science Review*, Volume 12, Issue 6, 2025, nwaf154, https://doi.org/10.1093/nsr/nwaf154), the erroneous notation ‘Nb 3*d*’ was used at page 1, line 23; page 2, line 39; page 4, lines 1 and 5; page 5, line 3, as well as page 9, lines 17 and 22.

Based on the electron configuration of Nb (4*d*^4^5*s*^1^), the valence *d*-orbital of Nb corresponds to the 4*d* orbital instead of the 3*d* orbital. All the aforementioned instances of ‘Nb 3*d*’ shall be revised to ‘Nb 4*d*’.

This constitutes a conceptual notation error only, with no impact on the experimental data, core conclusions, or the logical framework underpinning the material’s mechanism.

