# Supplementary material for: TT-Nb2O5 with dual-band modulation and exceptional performance retention for fast-responsive electrochromics
Source: Natl Sci Rev. 2025 Apr 24;12(6):nwaf154. doi: 10.1093/nsr/nwaf154 (PMC12139003; doi:10.1093/nsr/nwaf154)
Supplement: nwaf154_Supplemental_File [file nwaf154_supplemental_file.pdf]

Supplementary information for

TT-Nb_2O_5 with dual-band modulation and excellent performance retention for fast-responsive electrochromics

Qingjiao Huang[1], Peipei Shao[1], Mingao Hou[1], Yihan Lei[1], Zhexuan Ou[1], Jiacheng Hu[1], Ying Zhu[1], Bowen Li[1], Menghan Yin[1], Yiwen Zhang[1], Renfu Zhang[1], Jiawei Sun[1], Changjian Li[1], Guangfu Luo[1,3], Rui-Tao Wen[1,2,] *

[1]Department of Materials Science and Engineering, Southern University of Science and Technology, Shenzhen 518055, China

[2]Guangdong Provincial Key Laboratory of Functional Oxide Materials and Devices, Southern University of Science and Technology, Shenzhen, 518055, China

[3]Institute of Innovative Materials, Southern University of Science and Technology, Shenzhen 518055, China.

This file includes:

Materials and Methods

Supplementary Figures 1 to 27

Supplementary Note 1-3

Supplementary Table 1-4

Supplementary References 1-23

Materials and Methods

Thin films deposition and annealing

The Nb_2O_5 thin films were prepared by reactive direct current (dc) magnetron sputtering using a pure niobium target with diameter of 76.2 mm (purity: 99.9%). The ITO coated quartz glass (6 ohm/sq, South China Xiangcheng Technology Co., Ltd) was used as substrate. Meanwhile, borosilicate glass (7-10 ohm/sq, Guluo Glass Co., Ltd) coated with ITO was also used to obtain TT-Nb_2O_5 where it was found the borosilicate glass is stable after annealing at 600°C for two hours in air. It was found the electrochromic performance of TT-Nb_2O_5 is independent of the employed ITO/quartz and ITO/borosilicate glass. The deposition of Nb_2O_5 was conducted in a mixture atmosphere of Ar and O_2 in the ratio of 45:5, a working pressure of 1.0 Pa and a dc power of 150 W. The substrate was maintained at room temperature and kept rotating at 10 rpm to ensure film uniformity. The thickness of Nb_2O_5 films was in the range of 350±10 nm. The as-deposited Nb_2O_5 films were amorphous. To obtain TT- and T- Nb_2O_5, thermal annealing of the amorphous Nb_2O_5 (*a*-Nb_2O_5) films were conducted under 600℃ and 850℃ for 2 h in air, respectively. To ensure a parallel comparison of *a*-, T- and TT-Nb_2O_5, we also annealed ITO/quartz substrate at 600℃ for 2 h to ensure the identical resistance ITO electrode before *a*-Nb_2O_5 deposition.

A series of other cathodic electrochromic oxides (*i.e.*, WO_3, TiO_2, MoO_3, V_2O_5, $Nb_{18}W_{16}O_{93}$) were also prepared by reactive dc magnetron sputtering, with the aim to compare the diffusion coefficient and electrochromic responses with TT-Nb_2O_5. The metallic target was a 3-inch-diameter plate of the associated metals, for example, tungsten/molybdenum/vanadium/titanium (purity: 99.999%). $Nb_{18}W_{16}O_{93}$ was prepared through co-sputtering with tungsten (W) and niobium (Nb) targets. All the annealing were conducted in air. The detailed deposition and annealing parameters are shown in **Table S1** below.

The ion storage layer was prepared through co-sputtering with niobium (Nb) and vanadium (V) targets in an O_2/Ar mixture atmosphere with a ratio of 5:45, during which the sputtering pressure were kept at 1.2 Pa. The sputtering power applied to the Nb and V targets were set at 100 W and 150 W, respectively, in order to achieve a 1:1 atomic ratio of Nb and V in the niobium vanadium oxide ($NbVO_5$) thin films. The thickness of the $NbVO_5$ films was approximately 300 ± 10 nm. Deposition of NiO_x film was performed in a mixture of O_2 (99.99%) and Ar (99.99%) with a mass

flow ratio of 10%, during which the sputtering pressure and power were kept at 2.0 Pa and 200 W. The thickness of the NiO_x films was approximately 300 ± 10 nm.

Table 1. The preparation of cathodic electrochromic oxides

Films	Pressure (Pa)	Ar: O_2 (Sccm)	Power (W)	Thickness (nm)	Annealing temperature (℃)	Annealing time (h)	Phase
WO_3	4.0	70:10	200	300±10	400	2h	Monoclinic
MoO_3	4.0	55:5	150	300±10	400	2h	Monoclinic
TiO_2	1.0	60:2.5	200	300±10	400	2h	Tetragonal
V_2O_5	1.0	45:5	150	300±10	350	2h	Orthorhombic
$Nb_{18}W_{16}O_{93}$	1.0	60:10	200(Nb):75(W)	300±10	850	1h	Orthorhombic

Fabrication of the full electrochromic devices

The full devices utilized TT-Nb_2O_5 and $NbVO_5$ as the working and counter electrode, respectively. Initially, the TT-Nb_2O_5 and $NbVO_5$ films were glued together with a face-to-face configuration. The distance of the two electrodes were 1mm, separated by spacers. The electrolyte was $LiClO_4$ dissolved in propylene carbonate (PC) with a concentration of 1 mol L^{-1}. Then, the electrolyte was mixed with the acrylate-based UV curable glue in a volume ratio of 2:1 and thoroughly stirred for 30 minutes under complete light shielding. The prepared solution was injected into the device using a syringe until the space was completely filled. Finally, the device was cured by placing it under a UV lamp (Philips, 9 W, 365 nm) for 20 minutes. After simple packaging, the device structure can be described as Glass/ITO/TT-Nb_2O_5/polymer electrolyte/$NbVO_5$/ITO/Glass. The full WO_3/NiO_x devices used for comparison of energy savings are assembled through the above process. The full devices feature a five-layer sandwich configuration, namely Glass/ITO/WO_3/polymer electrolyte/NiO_x/Glass.

Characterization

Crystalline structure of Nb_2O_5 thin films were characterized by X-ray diffractometer (Smartlab, Rigaku) at an angle of 1.5 ^{o} under grazing incidence mode with a Cu K*a* radiation source

(λ=0.15418 nm). All the as-deposited oxides were X-ray amorphous. The crystalline phases were obtained through annealing as described above. The crystal structure of TT-Nb_2O_5 was determined using a Crystallographic Information File (CIF) from the Cambridge Crystallographic Data Centre (CCDC) with #2103847. The crystal structure of T-Nb_2O_5 was constructed using a CIF from the Inorganic Crystal Structure Database (ICSD) with #1840.

Transmission electron microscope (TEM; Tecnai F30, FEI, USA) combined with focused ion beam (FIB) to confirm the crystalline structure of Nb_2O_5 thin films. Meanwhile, the crystalline structure of TT-Nb_2O_5 thin films was further corroborated by scanning transmission electron microscopy (STEM; Titan ETEM G2) combined with FIB milling. Firstly, a protective carbon layer was deposited on the surface of the target region of the sample to prevent damage during the milling process. Subsequently, the FIB was employed to cut out a lamella with a thickness of approximately 50 nm. The prepared samples were then carefully transferred onto copper grids for further examination. The chemical composition and valences of Nb_2O_5 thin films were characterized by X-ray photoelectron spectroscopy (XPS). XPS was carried out using a Escalab Xi^+ from Thermo Fisher Scientific equipped with a monochromatic Al Kα radiation source ($h\nu$ = 1486.7 eV). For X-ray photoelectron spectroscopy (XPS) analysis, the samples $Li_xNb_2O_5$ at different potentials were transferred from a glove box to the XPS chamber by using a vacuum transfer chamber, effectively avoid the oxidation and contamination. The samples were cleaned with dimethyl carbonate (DMC, Sigma-Aldrich, >99.5%) to remove the surface residuals. Raman spectra of Nb_2O_5 films under different states were collected by a Horiba LabRAM Aramis instrument using a 50 × microscope objective and an excitation wavelength of 532 nm.

Electrochemical and *in situ* optical measurements

The electrochromic performance of Nb_2O_5 thin films and other electrochromic oxides were conducted in glove box. The glove box is filled with Ar atmosphere and the concentration of water and oxygen is less than 0.01 ppm. The electrochromic properties were evaluated in a three-electrode system with Li foils as reference and counter electrodes. Nb_2O_5, V_2O_5, WO_3, TiO_2, MoO_3 and $Nb_{18}W_{16}O_{93}$ thin films were taken as working electrode and $LiClO_4$-PC (1 mol L^{-1}) as electrolyte. Cycling voltammetry (CV), galvanostatic intermittent titration technique (GITT) and electrochemical impedance spectroscopy (EIS) and chronoamperometry (CA) were conducted on

an electrochemical workstation (Iviumn stat). Optical transmittance spectra were recorded in real-time by a fiber-optical instrument from Ocean Optics (QEpro, Ocean Optics, USA). The effective optical range was from 380 to 2250 nm. The in-situ reflectance spectrum of TT-Nb_2O_5 was recorded through a reflection probe from Ocean Optics in the same spectra range (QEpro, Ocean Optics, USA). For TT-Nb_2O_5, the reversible potential range was explored by comparing two low cut-off potentials, as shown in **fig. S7**. The full device measurements were carried out as a two-electrode cell in glove box. During the chronoamperometry measurement, in-situ transmittance of the full devices was monitored using a fiber-optic instrument by Ocean Optics (QEpro, Ocean Optics, USA).

Table 2. The voltage range of cyclic voltammetry for cathode electrochromic oxides.

**Films**	WO_3	MoO_3	TiO_2	V_2O_5	$Nb_{18}W_{16}O_{93}$	Nb_2O_5
Amorphous	2.0-4.0 V	2.7-4.7 V	1.25-4.0 V	2.5-4.5 V	/	1.2-3.5 V
Crystalline	2.0-4.0 V	2.7-4.7 V	1.25-4.0 V	2.5-4.5 V	1.5-3.5 V	1.2-3.5 V

Lithiation calculations

The density of Nb_2O_5 is documented as 4.6 g/cm^3. To calculate the number of inserted Li in TT-Nb_2O_5 during the CV cycling, the standard density of Nb_2O_5 is multiplied by a factor of 0.8, by considering the potential porosity of the obtain crystalline Nb_2O_5 through sputtering process.[1]

Lattice constant and volume of TT-Nb_2O_5 under various Li concentrations

The lattice constant and volume of a unit cell evolved with Li concentrations was analyzed from XRD pattern, employing an equation written as:

$$d_{hkl} = \frac{1}{\sqrt{\frac{4}{3}\frac{(h^2+hk+k^2)}{a^2}+\left(\frac{l}{c}\right)^2}}$$

$$V = a^2 c sin(120°)$$

Where *d* is crystalline inter-planar spacing, *a*, *b* and *c* are lattice constant, *h*, *k* and *l* are the Miller indices of crystallographic plane. *V* is the volume of a unit cell.

Computational details on first-principles calculations

The first-principles calculations in this work are based on the density functional theory as implemented in the Vienna Ab-initio Simulation Packag[2]. The generalized gradient approximation in the Perdew-Burke-Ernzerhof[3] form is chosen as the exchange-correlation functional for most calculations, unless stated otherwise. The Hubbard U method[4-6] with an effective U value of 4.0 eV[7] is applied to the 3*d* electrons of Nb atoms. The accuracy of this PBE+U method is validated by comparing the density of states of pristine TT-Nb_2O_5 with that obtained from the hybrid exchange-correlation functional HSE06[8], as shown in **fig. S4**. A plane-wave energy cutoff of 339 eV is employed, along with the following projector augmented wave pseudopotentials: Li_PBE ($2s^1$) for lithium, Nb_PBE ($4p^6 5s^1 4d^4$) for niobium, and O_PBE ($2s^2 2p^4$) for oxygen. A Monkhorst-Pack mesh with a *k*-point spacing of ~2π/60 $Å^{-1}$ is adopted. The energy and force tolerances are set to 10^{-5} eV and 0.02 eV/Å, respectively. The initial crystalline structure of TT-Nb_2O_5 is obtained from the Cambridge Crystallographic Data Centre (CCDC #2103847) and a stoichiometry of Nb:O = 2:4.75 is determined to reproduce the experimental XRD result for the DFT-optimized structure. The supercells of $Li_xNb_2O_5$ (x = 0.31, 0.77, and 1.32) contain 113–129 atoms.

Supplementary Note 1: diffusion validation

The charge exchange features of Nb_2O_5 with different polymorphs are analyzed through the formula:[9]

$$i_p = \mathrm{C}v^b$$

where i_p is the peak current density in A cm^{-2}, v is the sweep rate in mV s^{-1}, C is a constant. The features of Li ion pseudo-capacitive intercalation were reported and detailed documented in ref.[10]. When b=1, there are predominantly intercalation pseudo-capacitive reactions taking place. As b is between 0.5 and 1, the electrochemical reaction is a combination of pseudo-capacitance and ion bulk diffusion. The value of b for TT-, a- and T-Nb_2O_5 are 1.0, 1.0 and 0.7, respectively **(fig. S11)**, suggesting a pseudo-capacitive intercalation in TT-Nb_2O_5 **(fig. S12)**. The primary factor contributing to the identification of intercalation pseudo-capacitive behavior in TT-Nb_2O_5 is ascribed to three distinct attributes. *i*) The peak currents scale a linear relationship with v. *ii*) The atomic structure of TT-Nb_2O_5 exhibits a two-dimensional (2D) pathway that enables efficient

diffusion of Li ions. Moreover, the ion transport process does not involve a phase transition; but a solid solution process **(Fig. 2j-l)**. *iii*) The capacity remains relatively stable independent of the charging time **(fig. S11)**.

Supplementary Note 2: electrochemical kinetic analysis

To further verify the electrochemical kinetic characteristics of different polymorphs of Nb_2O_5 and other cathodic electrochromic oxides, GITT measurements were carried out with a constant current pulse of 0.2 mA for 10 s, followed by a 30 s rest period. The specific information of GITT is shown in **fig. S18-20**. Li-ion diffusivities (D_{Li}^{+}) can be confirmed by extrapolating from the equation:

$$D_{Li^+} = \frac{4}{\pi\tau}(\frac{m_B V_m}{M_B A})^2(\frac{\Delta E_S}{\Delta E_\tau})^2, \qquad \tau \ll \frac{L^2}{D_{Li^+}}$$

Where the $\mathbf{V_m}$ and $\mathbf{M_B}$ are the molecular molar volume and mass, ***A*** is the area of active electrode immersed in electrolyte. τ is the duration time of the constant pulse and *L* is the thickness of electrode. ΔE_τ and ΔE_s are the potential difference.

Furthermore, electrochemical impedance spectroscopy (EIS) has also been utilized for the assessment of the Li-ion diffusion coefficient due to its ability to provide kinetic data that can be correlated with the diffusion state. This measurement is carried out by applying a low-amplitude signal near an equilibrium state at open circuit state within the frequency range of 0.01–100,000 Hz and accompany with small disturbance voltage about 10 mV. *Nyquist* plot of each cathodic electrochromic oxide exhibits a high-frequency semicircle and a low-frequency Warburg tail region **(fig. S22a)**. The high-frequency semicircle is indicative of charge-transfer resistance associated with interfacial Li-ion transfer, while the Warburg tail region is linked to Li-ion diffusion within the active material. The real component of the resistance (*Z*') is plotted against the inverse square root of the angular speed (ω) in the low-frequency range from 1 to 0.01 Hz as is shown in Supplementary **fig. S22b**. Warburg factor (σ) is derived from the slope. The lithium diffusion coefficient is deduced from the equation written as:

$$D_{Li^+} = \frac{R^2 T^2}{2A^2 n^2 F^4 C^2 \delta^2}$$

where ***R*** is the gas constant (8.134 J mol^{-1} k^{-1}), **T** is the absolute temperature (298.15 K), **F** is Faraday's constant (9.6468×10^4 C mol^{-1}) and ***C*** is the molar concentration of Li ions in an active

material, n is the number of electrons which is 1 in current case.

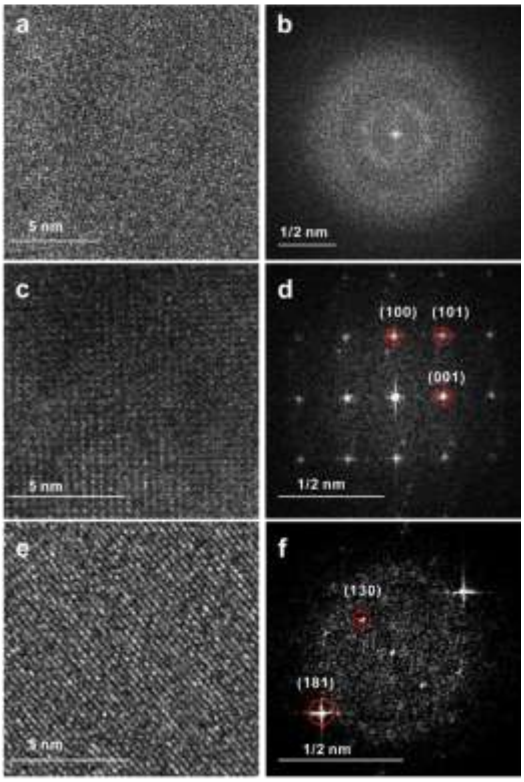

Figure S1. TEM images of Nb_2O_5 thin films with various polymorphs. The high-resolution TEM image and associated fast Fourier transform patterns of (**a-b**) *a*-Nb_2O_5, (**c-d**) TT-Nb_2O_5, (**e-f**) T-Nb_2O_5.

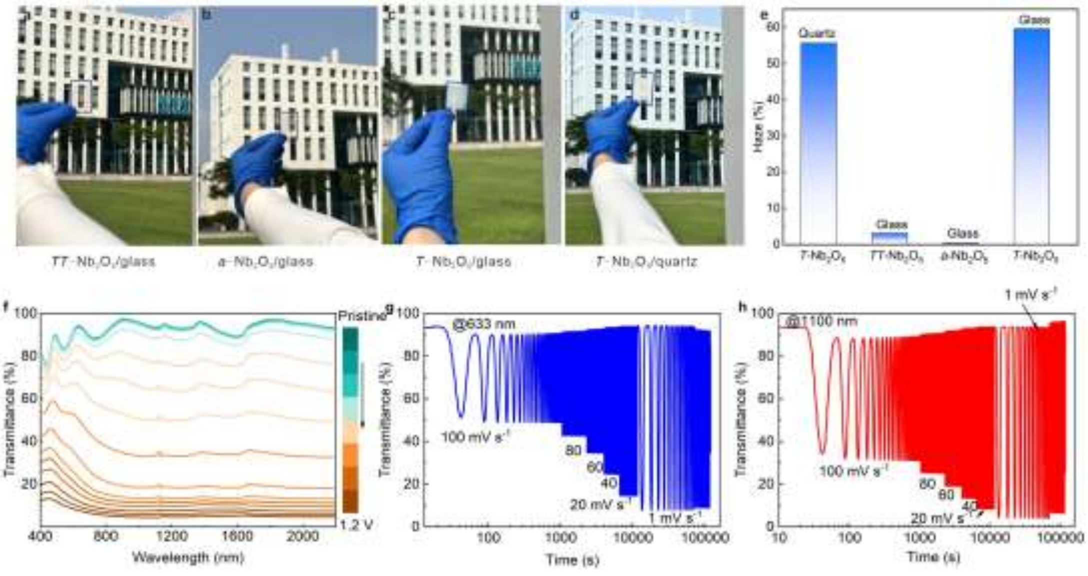

Figure S2. Electrochromic properties of TT-Nb_2O_5 thin films deposited on ITO/glass. (**a-d**), The digital images of Nb_2O_5 films with different phases. (**e**), The optical haze of Nb_2O_5 films with different phases. **(f)** The *in-situ* optical transmittance variation of TT-Nb_2O_5 thin films observed during the electrochemical testing process. The sweep rate was 1 mV s^{-1}, in a three-electrodes configuration. **(g-h)** Δ**T** as a function of various sweep rates for 633 nm (VIS) and 1100 nm (NIR) in TT-Nb_2O_5, respectively. The corresponding sweep rate was marked.

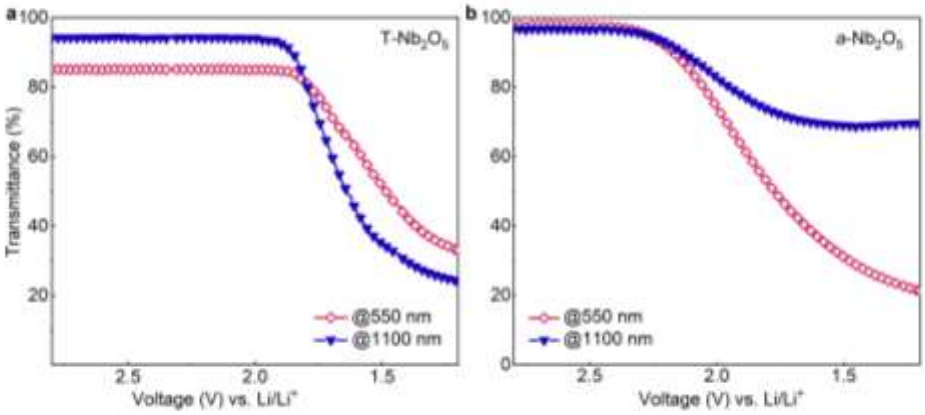

Figure S3. (a-b) Optical modulation of T- and *a*-Nb_2O_5. Optical data is recorded during CV cycling which were conducted in the potential range of 1.2-3.5 V at a sweep rate of 1 mV s^{-1}.

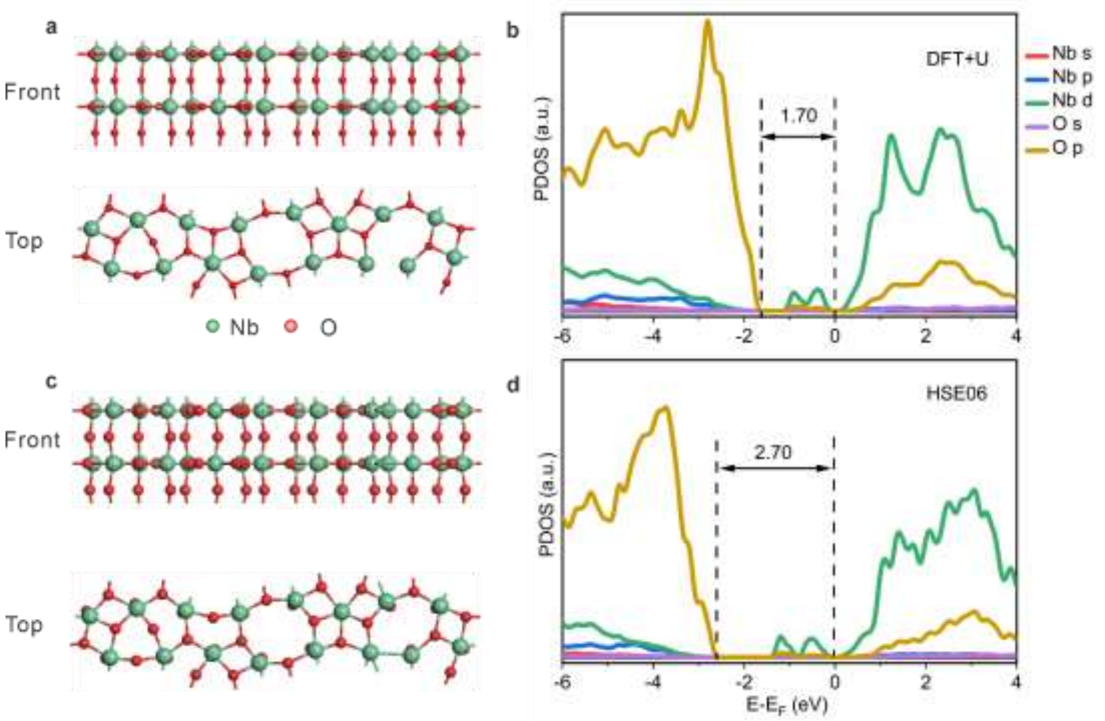

Figure S4. (a) Optimized structure and **(b)** projected density of states (PDOS) for TT-Nb_2O_5 calculated by the PBE+U method; **(c)** Optimized structure and **(d)** PDOS for TT-Nb_2O_5 calculated by the HSE06 method. Except of the underestimated band gap by the PBE+U method, which is a well-known feature, the overall shapes of the PDOS are consistent between both methods.

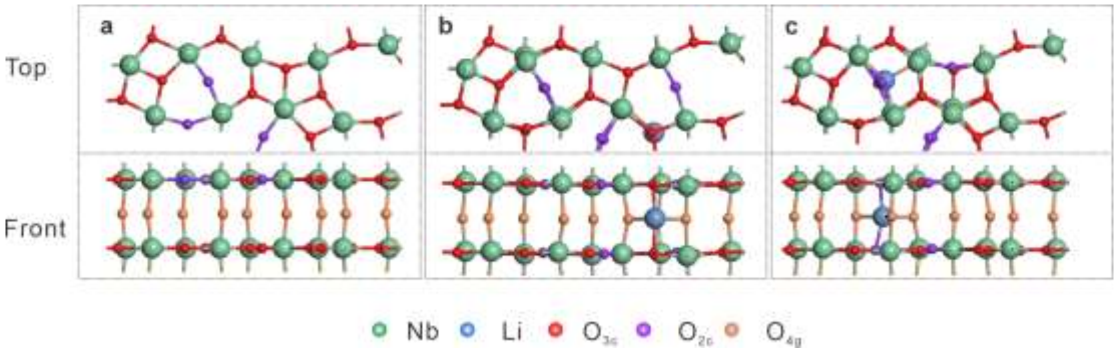

Figure S5. (a-c) Top and front views of pure TT-Nb_2O_5 and ones with intercalated Li bonding to O_{3c} or O_{2c} in the oxygen plane (4h). Within this structure, three types of oxygen surrounding are identified: O_{3c}, O_{2c} and O_{4g}. The Li atoms are found to preferentially occupy sites between O_{3c} and then sites between O_{2c}, as is consistent with the two experimental voltage plateaus observed (**Fig. 2h**).

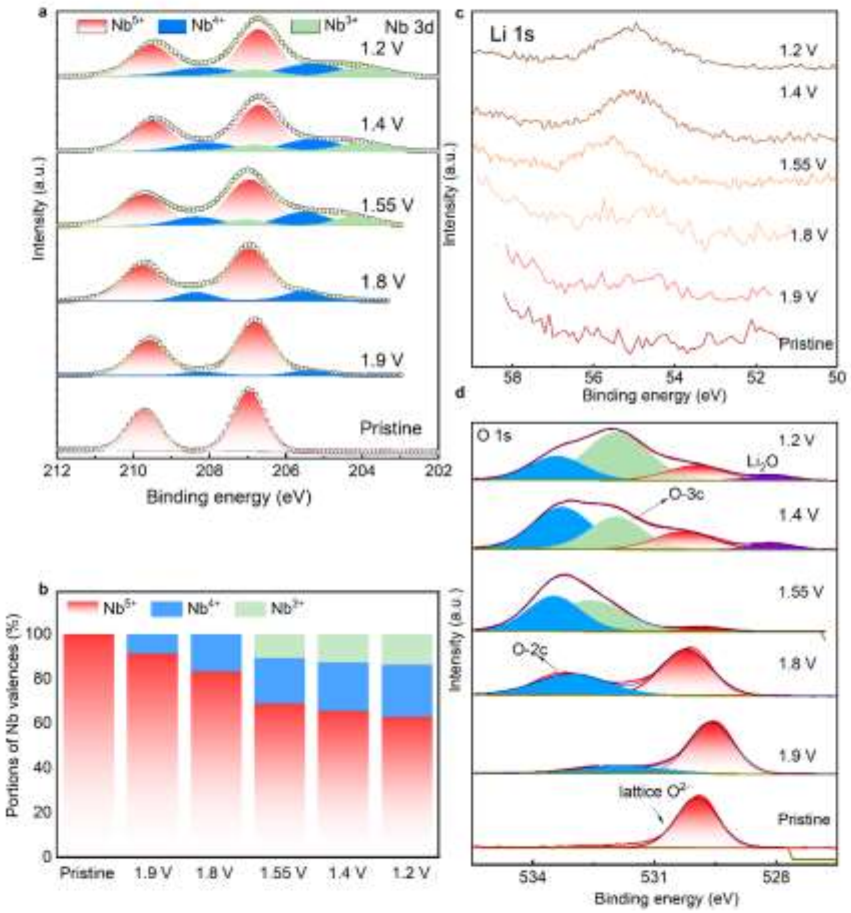

Figure S6. Composition and valency analysis of TT-Nb_2O_5 thin films. (a) and **(c)** The X-ray photoelectron spectroscopy (XPS) data of the Nb 3d Li 1s. **(d)** O 1s spectra of TT-Nb_2O_5 thin films at marked potentials. **(b)** Proportions of different Nb valences at marked potentials. At the pristine state, the pairing peaks arise from splitting spin orbit of Nb^{5+}[11, 12]. Upon ion insertion to 1.8 V where mainly the NIR is modulated, another pair-peak emerged and assigned to Nb^{4+}, consistent with the reduction from Nb^{5+} to Nb^{4+}[13-15]. Further ion insertion to 1.55 V leads to an appearance of a new pair-peak which corresponds to Nb^{3+}[16-19]. Upon charging to 1.2 V, the paired peaks remain unchanged except for the slightly increased ratio of Nb^{3+} and Nb^{4+} to Nb^{5+}. Associated signals of oxygen and lithium were consistent with Nb_{3d}. The strongly correlated Nb_{3d} valency and optical spectra certainly bonds to the varied optical modes, as revealed by our DFT calculations.

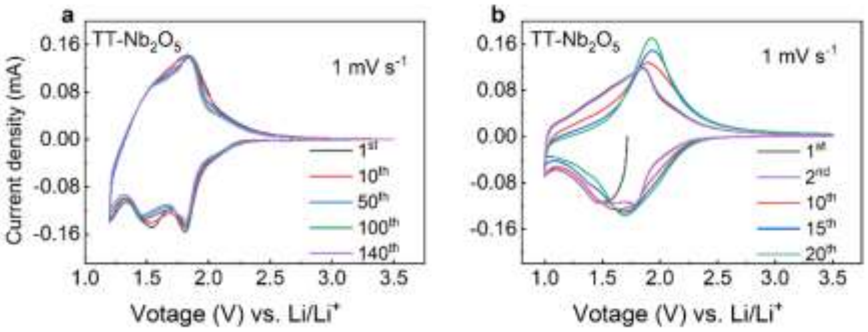

Figure S7. Electrochemical characteristics of TT-Nb_2O_5 operated within various voltage ranges for reversible potential range assessment. (a) 1.2-3.5 V; **(b)** 1.0-3.5 V, relative to Li/Li^+ reference electrode, using a scan rate of 1 mV s^{-1}.

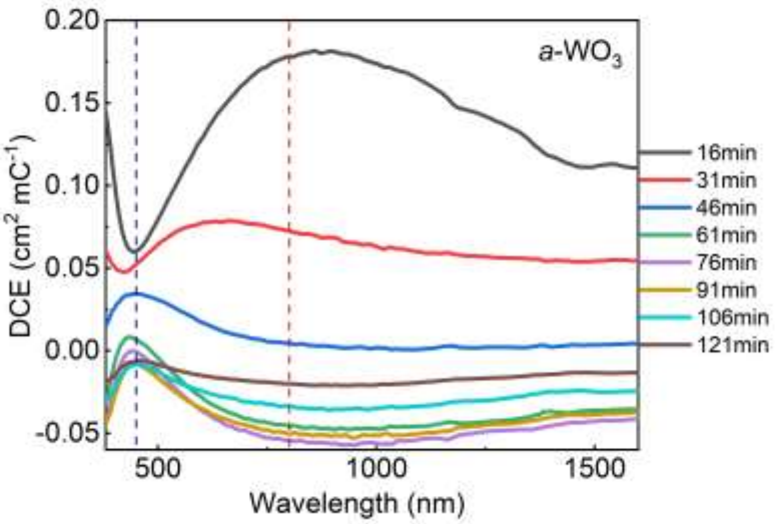

Figure S8. The differential coloration efficiency versus wavelength in an *a*-WO_3 thin film upon charge insertion. The measurements were conducted by loading a constant current of 3 μA cm^{-2}. It can be observed that the optical absorption peak centered at 800 nm initially, which gradually switched to 450 nm as current loading proceeded, indicating a polaron hopping transition from W^{6+}↔W^{5+} to W^{6+}↔W^{4+}, as marked by the two dotted lines.

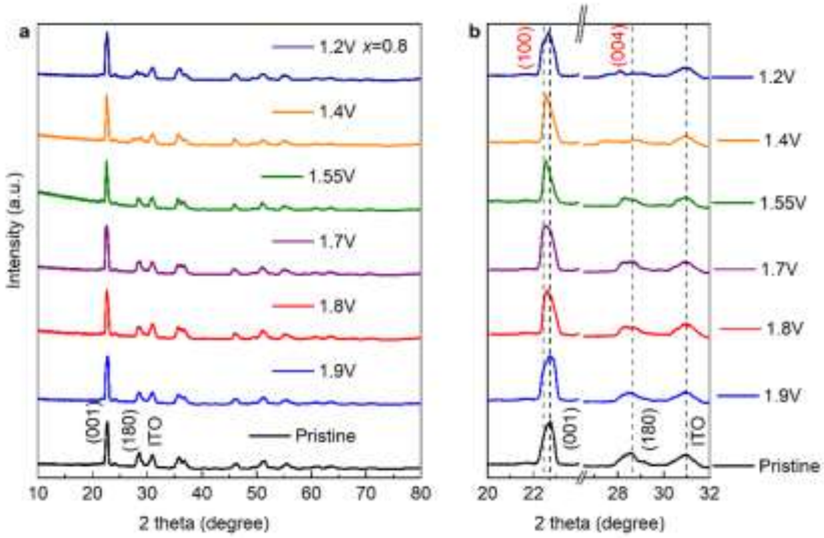

Figure S9. (a) *Ex-situ* XRD patterns of T-Nb_2O_5 film at the pristine and different states. (b) Enlarged view of (001) and (180) planes of T-Nb_2O_5. The unchanged position of ITO peak is intently included as a reference. When the potential drops to 1.20 V, two distinct diffraction peaks emerge and indexed as the (100) and (004) plane of monoclinic-phase Nb_2O_5 (JCPDS: 43-1042). This indicates that when the potential drops to 1.2 V (x=0.8), the T- Nb_2O_5 undergoes a phase transition from orthogonal to monoclinic.

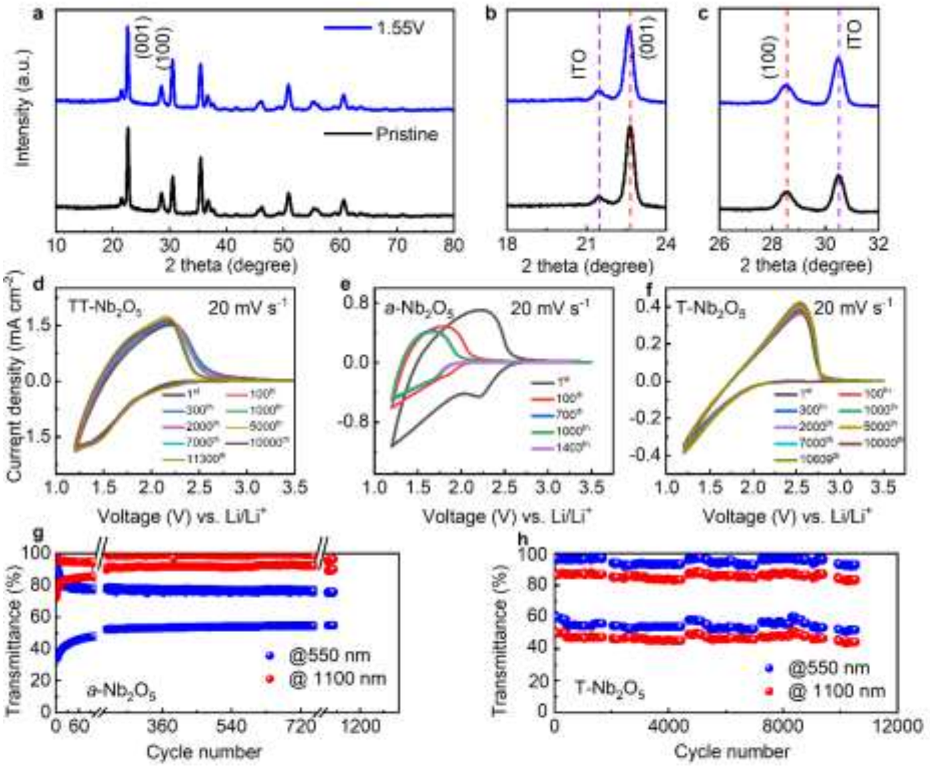

Figure S10. Long-term cycling of Nb_2O_5 thin films with various polymorphs. (a) Ex situ XRD patterns of TT-Nb_2O_5 film at marked potentials, representing different Li concentrations with a sweep rate of 20 mV s^{-1}. (**b-c**) Enlarged view of (001) and (100) planes of TT-Nb_2O_5. The unchanged position of ITO peak is intently marked as a reference. (**d-f**) CV curves of TT- Nb_2O_5, T-Nb_2O_5 and *a*-Nb_2O_5 films. The CV was conducted in potential range of 1.2 - 3.5 V vs Li/Li^+ at a sweep rate 20 mV s^{-1}. It can be observed that TT- and T-Nb_2O_5 are very stable in the potential range, where *a*-Nb_2O_5 showed rapid degradation due to ion trapping. (**g-h**) Optical modulation of *a*-Nb_2O_5 and T-Nb_2O_5 thin films upon long-term cycling as shown in Figure E-F.

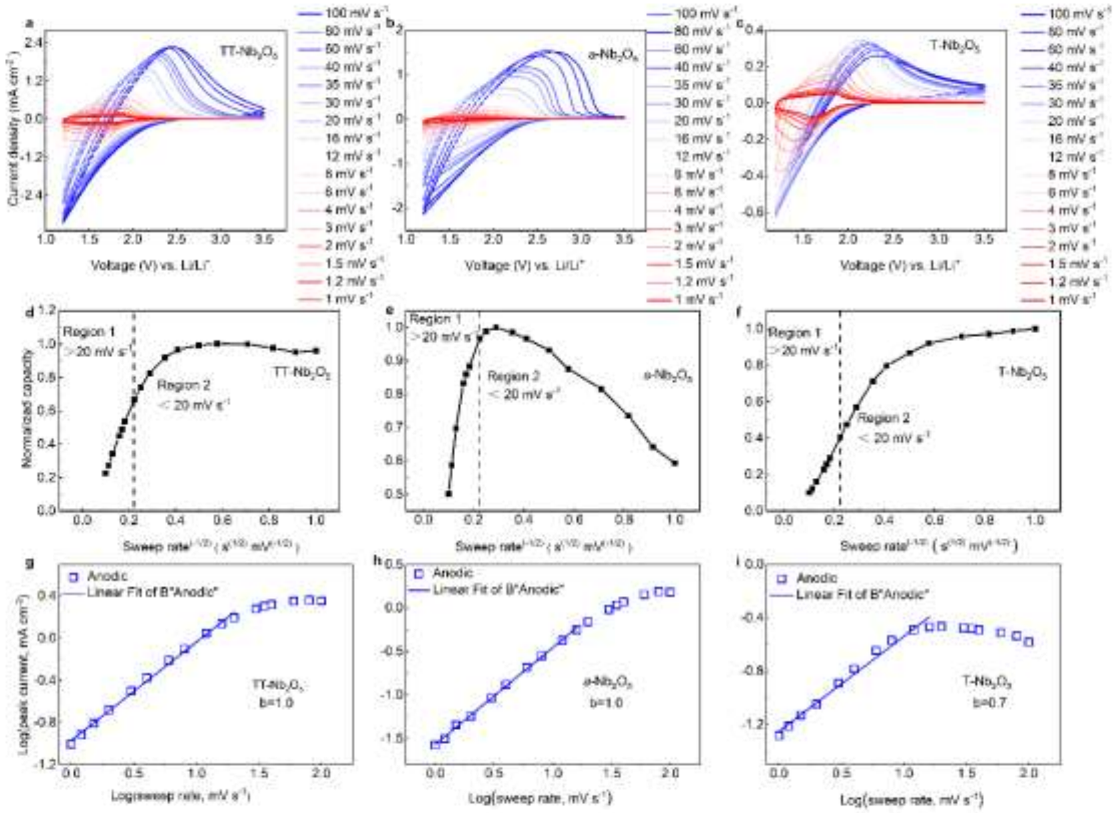

Figure S11 Kinetic analysis of TT-Nb_2O_5, *a*-Nb_2O_5 and T-Nb_2O_5. The CV curves of Nb_2O_5 thin films with various polymorphs. Cyclic voltammograms from 1 to 100 mV s^{-1} for TT-Nb_2O_5 (**a**), *a*-Nb_2O_5 (**b**) and T-Nb_2O_5 (**c**) to assess the electrochromic responses. The diffusion coefficient of Li ions was calculated by using the anodic peak current density of the CV curves at a sweep rate of 20 mV s^{-1}. The ion diffusion behavior and capacitor behavior are analysis through the variation tendency of normalized capacity evolution with root mean square of sweep rate. When the sweep rate is lower than 20 mV s^{-1}, it is observed that the capacity of TT-Nb_2O_5 remains relatively stable as the sweep rate lowers (**d**). In contrast, the capacity of T-Nb_2O_5 exhibits a gradual increase over time (**f**). Notably, ion trapping is observed in the *a*-Nb_2O_5 (**e**). *b*-value shows that it is approximately 1.0, 0.7, 1.0 for TT-Nb_2O_5 (**g**), *a*-Nb_2O_5 (**h**) and T-Nb_2O_5 (**i**) up to 20 mV s^{-1}, respectively.

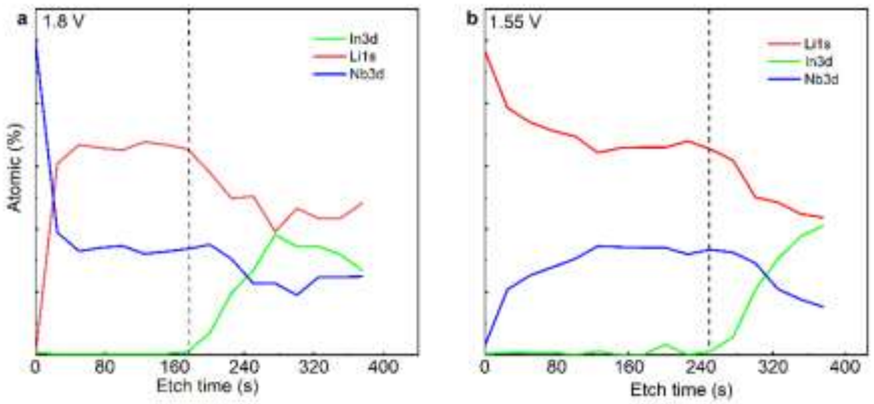

Figure S12 Depth profile of the element analysis in TT-Nb_2O_5 thin films under different reduction potentials. The reduction potentials of 1.8 V and 1.55 V are corresponding to the modulation of NIR and VIS, respectively. **(a)** data were taken at 1.8 V; **(b)** data were taken at 1.55 V. The dash line shows in was detected from ITO/glass. The results indicated that the incorporated Li ions were in a manner of diffusion, rather than surface process.

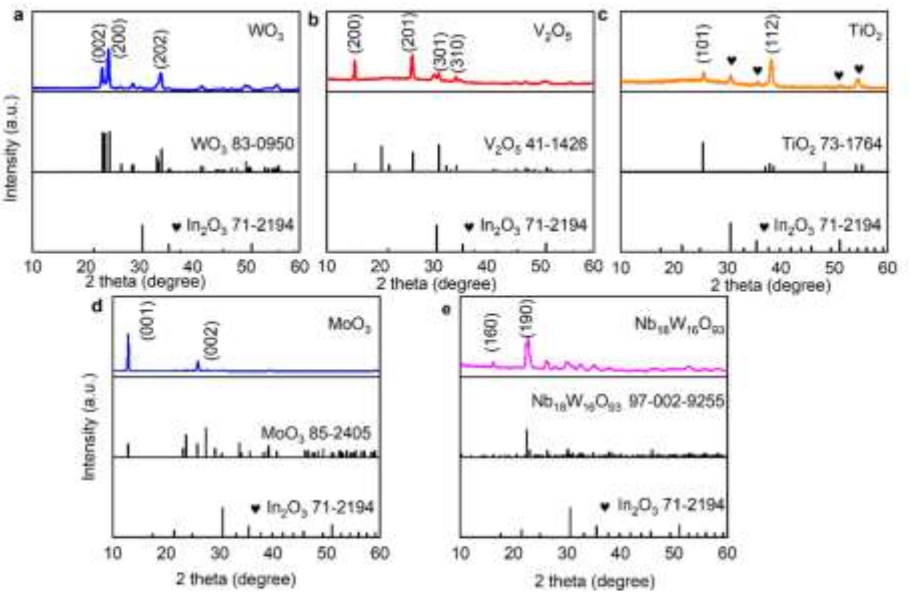

Figure S13 XRD characterizations of the cathodic electrochromic oxides. (a-e) XRD patterns of the crystalline of WO_3, V_2O_5, TiO_2, MoO_3 and $Nb_{18}W_{16}O_{93}$, respectively. The as-deposited WO_3, MoO_3, and TiO_2 thin films were annealed at 400℃ for 2 h. The as-deposited V_2O_5 thin film was annealed at 350℃ for 2h. The as-deposited $Nb_{18}W_{16}O_{93}$ thin films were annealed at 850℃ for 1h. The XRD results indicate that the oxides thin films demonstrate good crystallinity, matching well with the monoclinic (WO_3), monoclinic (MoO_3), orthorhombic (V_2O_5), anatase tetragonal (TiO_2) and orthorhombic ($Nb_{18}W_{16}O_{93}$) phases, respectively.

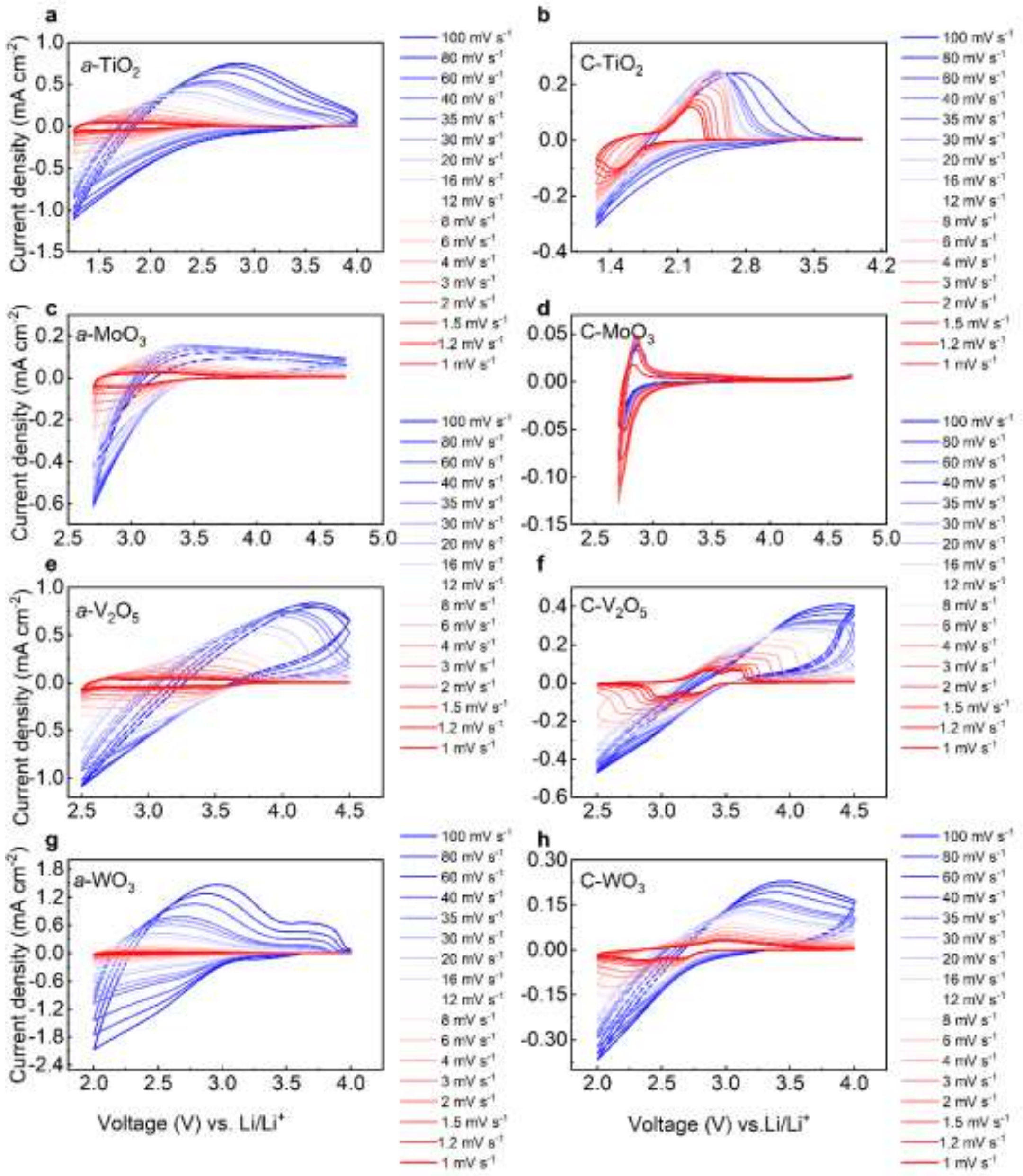

Figure S14 The CV curves of marked electrochromic oxide thin films at various sweep rates. **(a-b)** amorphous and crystalline TiO_2; **(c-d)** amorphous and crystalline $MoO_{3.}$ **(e-f)** amorphous and crystalline V_2O_5; **(g-h)** amorphous and crystalline WO_3. The anodic peak current density of the CV curves at a sweep rate of 20 mV s^{-1} was utilized for the calculation of the diffusion coefficient of Li ions.

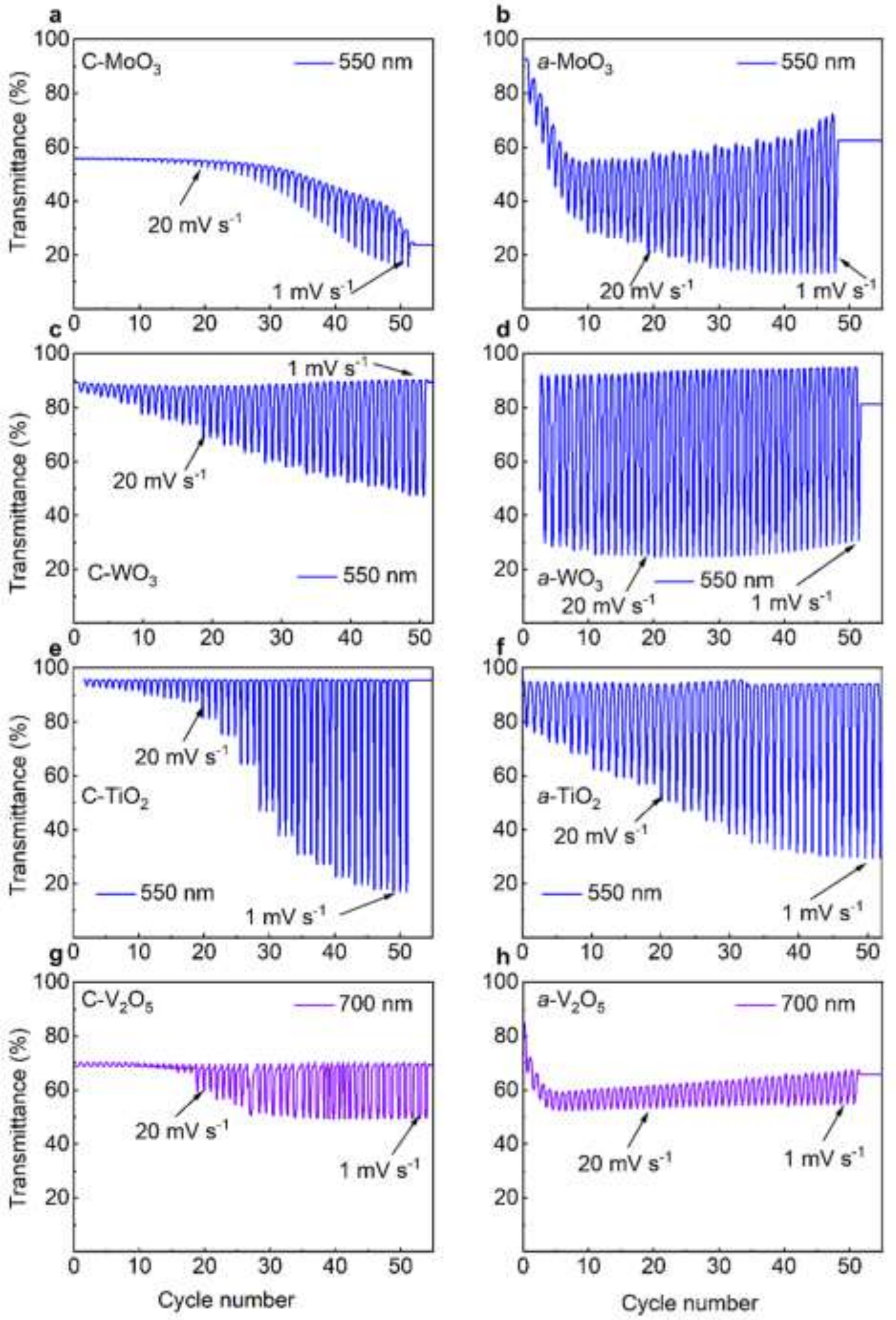

Figure S15 Optical transmittance variation of the marked electrochromic oxide thin films at the wavelength of 550 nm (VIS). The associated sweeping was shown in fig. S14 above. **(a-b)** crystalline and amorphous MoO_3; **(c-d)** crystalline and amorphous WO_3; **(e-f)** crystalline and amorphous TiO_2; **(g-h)** crystalline and amorphous V_2O_5. The fixed wavelength of 700 nm for V_2O_5 thin films is selected due to its absorption edge shifting below 550 nm.

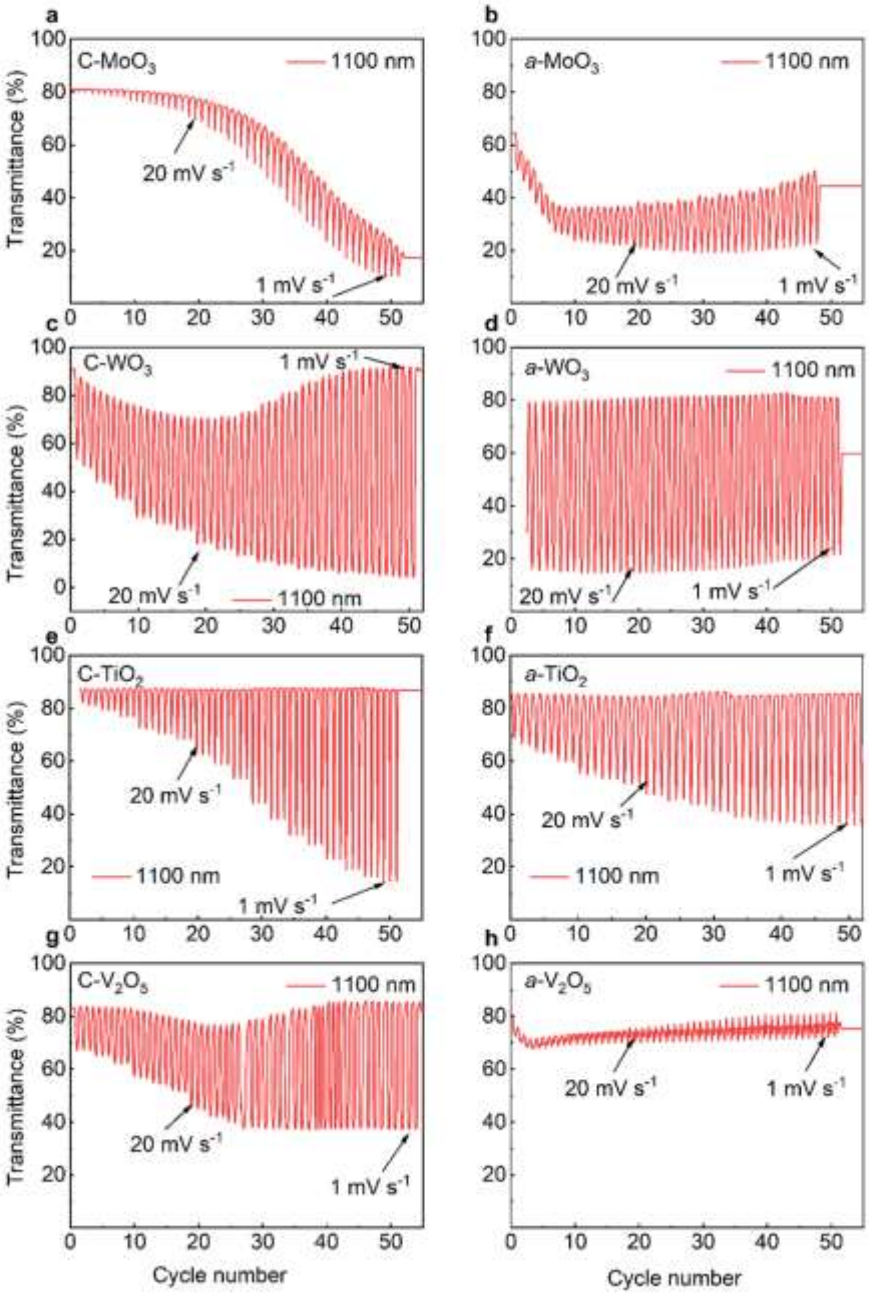

Figure S16 Optical transmittance variation of the marked electrochromic oxide thin films at the wavelength of 1100 nm (NIR). The associated sweeping was shown in fig. S14 above. **(a-b)** crystalline and amorphous MoO_3; **(c-d)** crystalline and amorphous WO_3; **(e-f)** crystalline and amorphous TiO_2; **(g-h)** crystalline and amorphous V_2O_5.

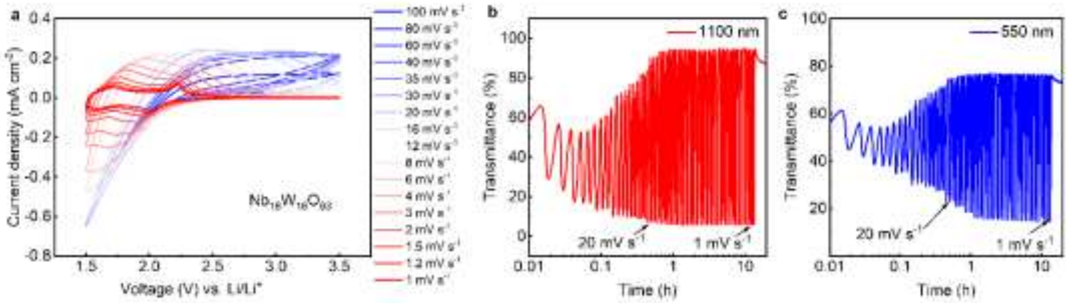

Figure S17 The electrochromic performance of $Nb_{18}W_{16}O_{93}$. (a) Cyclic voltammograms of $Nb_{18}W_{16}O_{93}$ were performed at a range of sweep rates, ranging from 1 to 100 mV s^{-1}. The anodic peak current density of the CV curves at a sweep rate of 20 mV s^{-1} was utilized for the calculation of the diffusion coefficient of Li ions, consistent with TT-Nb_2O_5 and other cathodic electrochromic oxides used in this paper. **(b-c)** Optical response of $Nb_{18}W_{16}O_{93}$ thin films at the wavelength of 550 nm (VIS) and 1100 (NIR). The optical switching at a fixed wavelength is in situ recorded during the process of CV cycling.

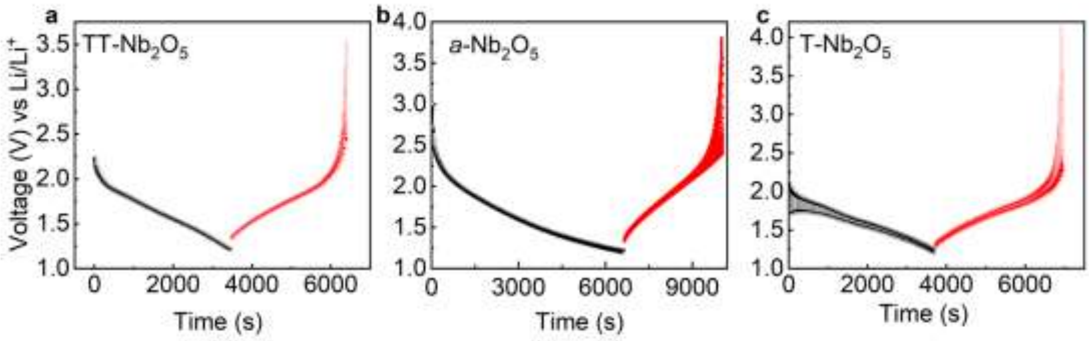

Figure S18 The GITT curves of TT-, *a*- and T-Nb_2O_5, respectively. For the GITT measurement, charging and discharging titrations were operated with the constant current pulse of 0.2 mA for10 s, followed by a 30 s rest. The single titration of GITT curves during the charge process was utilized for the calculation of the diffusion coefficient of Li ions.

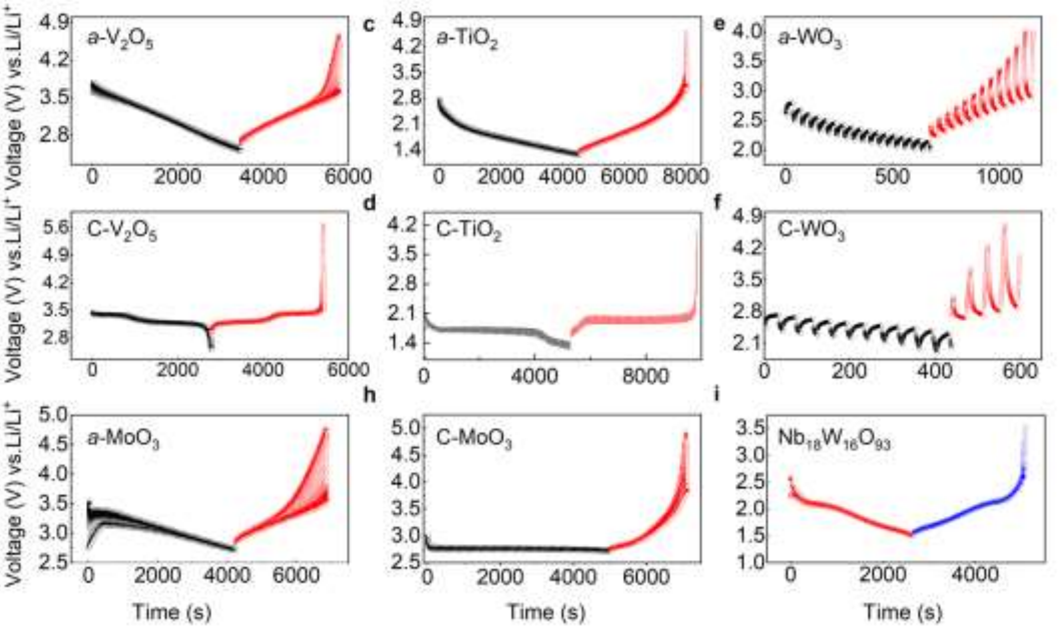

Figure S19 The GITT plots of the cathodic electrochromic oxides. (a-b) amorphous and crystalline V_2O_5; **(c-d)** amorphous and crystalline TiO_2. **(e-f)** amorphous and crystalline WO_3; **(g-h)** amorphous and crystalline MoO_3, **(i)** crystalline $Nb_{18}W_{16}O_{93}$. During GITT measurements, charging and discharging titrations were conducted using a constant current pulse of 0.2 mA for 10 s, followed by a 30 s rest. It is important to highlight that the constant current pulse for the GITT test of C-MoO_3 is 0.05 mA for 10 s, followed by a 30 s rest. The single titration of GITT curves during the charging process was utilized for the calculation of the diffusion coefficient of Li ions. The surface area of all samples immersed in the electrolyte was consistently maintained at 2.8 cm^2.

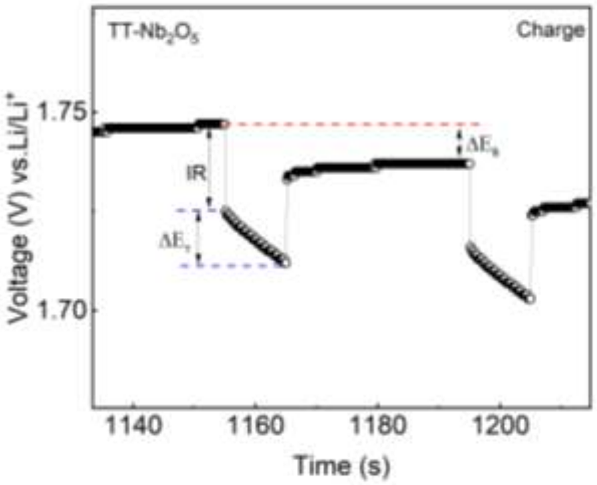

Figure S20 Enlarge view of the single titration from the GITT measurement at around 1.75 V (vs. Li^+/Li) during charging process for TT-Nb_2O_5.

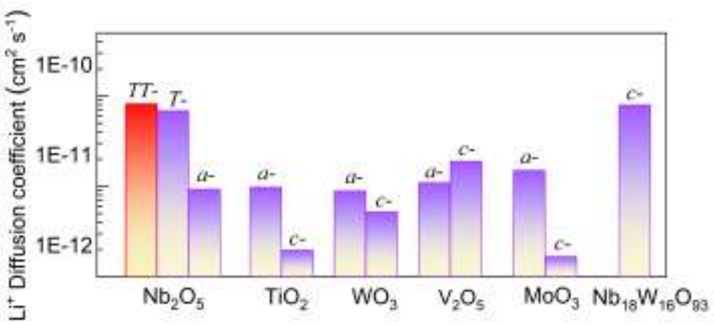

Figure S21 The diffusion coefficient of Li ions measured by GITT for *TT*-, *T*-, *a*-Nb_2O_5 and other cathodic electrochromic oxides. A singular titration within the GITT was employed to calculate the diffusion coefficient of lithium ions during the charging process.

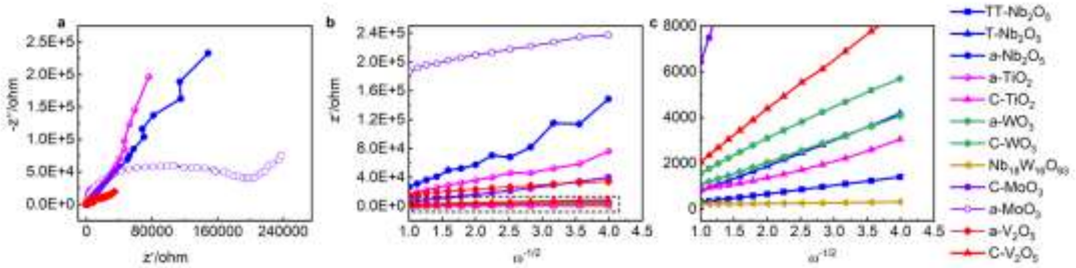

Figure S22 Li-ion diffusion coefficient analyzed by EIS. (a) EIS *Nyquist* plots of *TT*-, *T*-, *a*-Nb_2O_5 and other cathodic electrochromic oxides at a state of open circuit. **(b)** Z' vs $\omega^{-1/2}$ plots in the low-frequency range from 0.01 to 1.0 Hz. The Warburg factor (σ) is determined by the slope of the plots of Z' against the reciprocal square root of angular frequency ($\omega^{-1/2}$). **(c)** The enlarged view of the area framed with dotted black lines. The Warburg factor (σ) indicate the Li ion diffusion capability within electrochromic oxides. A smaller value of σ corresponds to a higher diffusion coefficient of the material, while a larger value of σ indicates a lower diffusion coefficient. The surface area of all samples immersed in the electrolyte was consistently maintained at 2.8 cm^2.

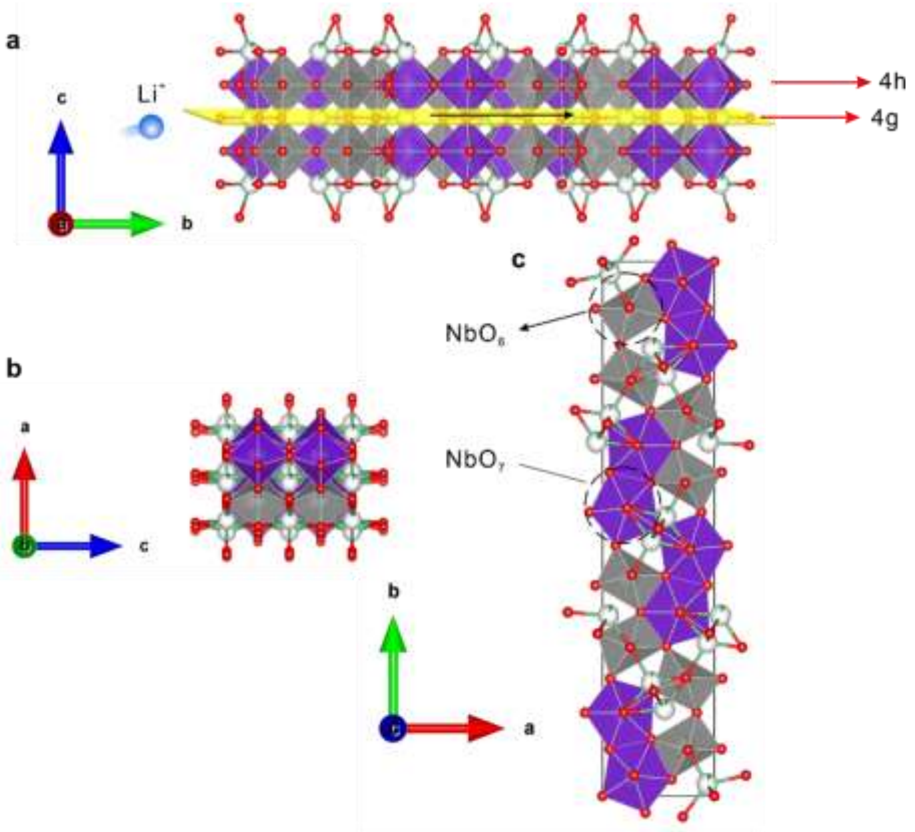

Figure S23 Crystal structure of *T*-Nb_2O_5. Schematic structures are viewing along a, b and c axis. The green and red spheres represent Nb and O atoms, respectively. The polyhedra marked with dashed circles denote distorted octahedra (NbO_6) and pentagonal bipyramids (NbO_7). The loosely and densely packed atomic layers are represented by 4g layer and 4h layer. It can be found that different from TT-Nb_2O_5, there is 0.8 Nb atom in 4*g* layer in a unit cell in T-Nb_2O_5. Distorted octahedra (NbO_6) and pentagonal bipyramidal (NbO_7) are also revealed in grey and purple.

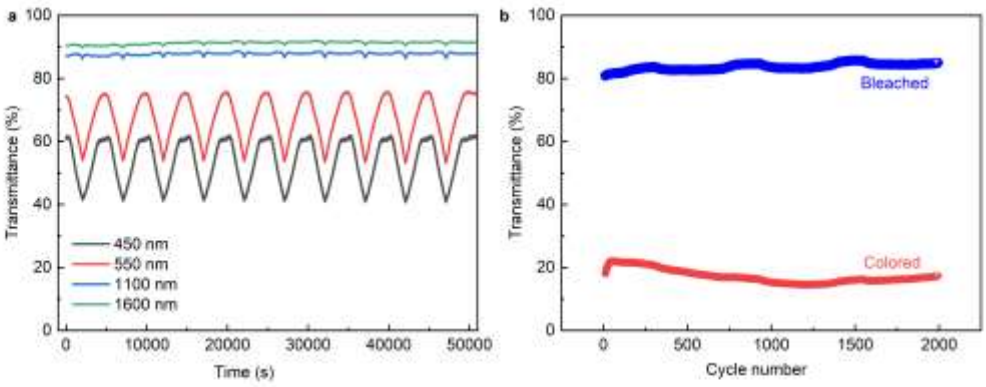

Figure S24. Electrochromic performance of $NbVO_5$ thin films (a) and cycle stability of TT-Nb_2O_5//$NbVO_5$ devices (b). (a) The *in-situ* optical transmittance at a fixed wavelength was recorded during cyclic voltammetry (CV) measurements, which were performed within the range of 1.5-4.0 V vs Li/Li^+ at a sweep rate of 1 mV s^{-1}. The results showed that $NbVO_5$ thin film is electrochromic inactive, which only served as an ion storage layer to match the dual band

modulation of TT-Nb_2O_5 layer in a full device. **(b)** The *in-situ* optical transmittance variation for a TT-Nb_2O_5//$NbVO_5$ devices was investigated at a fixed wavelength of 1100 nm during 2000 cycles of square-wave operation (100 s at 3.0 V for bleached state and 40 s at 4.0 V for colored state).

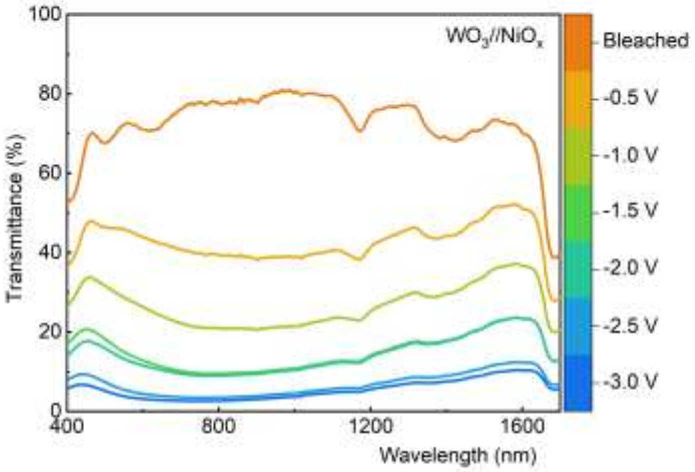

Figure S25 Electrochromic performance of full devices based on WO_3//NiO_x. As a state-of-the-art commercial in electrochromic field, we compared the energy savings of TT-Nb_2O_5/$NbVO_5$ device with them. The variation of full spectra (400 nm to 1700 nm) transmittance of device in respond to chronoamperometry (CA) testing were collected. CA measurement was performed within the potential range from 3.0 V to -3.0 V. The device exhibits a broad optical response from 400 nm to 1700 nm with the same optical evolution trend at 550 nm and 1100 nm.

Supplementary Note 3

We predicted the building energy consumption by EnergyPlus and OpenStudio software based on ASHARE 169-2013 standards[20] and visualize the predicted cooling energy saving pattern all over the world by ArcGIS Pro based on Koppen-Gieger climate zone classification.[21]

1. Building simulation model

In the simulation project, we created a medium office building (pre-1980) defined by Department of Energy (DOE) as the reference model shown in **fig. S26**. The office model has 3 floors with an aspect ratio of 1.5 (33.27×49.90 m^2) and glazing fraction of 33%. The total area is 4982 m^2 and the height between floors from bottom to top is 3.96 m, 3.96 m, 2.74 m. The building's north axis is 0 degree to true north. The thermostatic point is mainly set as $\boldsymbol{T}_h$ =21 ℃, $\boldsymbol{T}_c$ = 24 ℃ for heating mode and cooling mode in each simulation process. Corresponding

schedules are illustrated in **fig. S27**. The baseline cooling energy consumption pattern is established based on the reference window structure, double-pane 3.3 mm quartz glass spaced with 13 mm air. To predict cooling energy saving all over the world, other parameters are set as default in reference model other than components of exterior windows based on Nb_2O_5-$NbVO_5$ full device during simulation process.

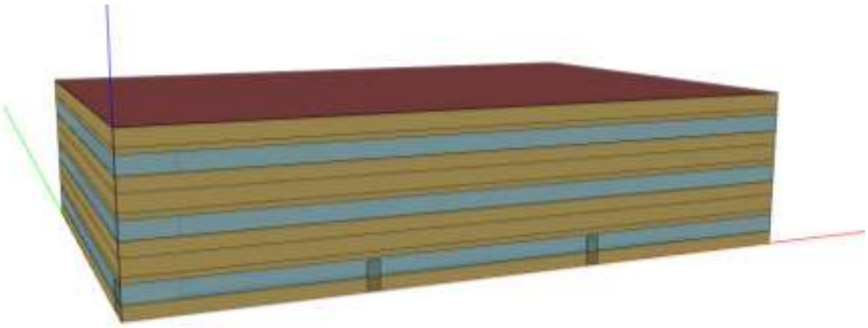

Figure S26 The illustration of medium office building geometry.

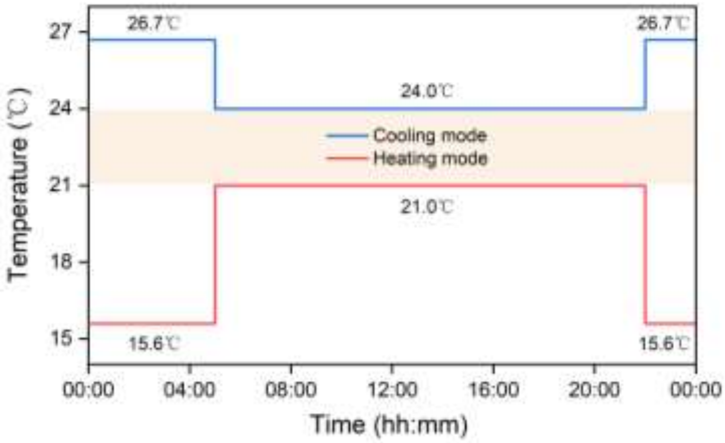

Figure S27 The corresponding schedules of thermostatic setpoint.

2. Climatic data and representative cities of climate zones

Ten cities were selected to represent all corresponding climate zones, as listed in Table 4 based on revised climate zone classification and their related climatic data applied in simulation process were downloaded from official website of EnergyPlus-Weather Data as the same for other 23 selected typical cities. These weather files contain typical hourly weather observations, similar to TMY format. It is often assumed that TMY data for these cities are representative of the entire

climate zone when performing large-scale analyses involving prototypical buildings.[22]

The cooling energy saving pattern of World map is visualized on the basis of KG climate zones and ASHARE Standard 169-2013 mainly. Due to the mismatch between Koppen-Geiger (KG) classification[22] and building energy simulation standards in EnergyPlus, it is necessary to map KG type to climate zones based on ASHARE Standard 169-2013 to facilitate the calculation, as shown in **Table 4**. Given data availability and integrity, we divided 30 KG climate zones into 10 categories to represent specific distribution levels of monthly average temperature in a typical year. Moreover, climates zones of selected 10 representative cities generally cover the entire levels from 0-8 based on ASHARE Standard.

Table 3: Mapping between two climate zone classification methods for the same 10 representative cities in building energy simulation project and their corresponding energy saving at both modes.

Climate zones A[21]	Representative cities	Climate zones B20	Cooling energy saving @ -3.0V and -5.5V (GJ)
**Af,** Am, Aw	Singapore	0A	86.82; 145.03
BSh, **BWh**	Alexandria, Egypt	2B	75.57; 114.93
**BSk**, BWk	Santiago, Chile	3C	56.69; 84.27
Csa, Cwa, **Cfa**	Shanghai, China	3A	76.21; 110.23
Csb, Cwb, **Cfb**	London, Britain	4A	83.21; 109.84
Csc, Cwc, **Cfc**	Auckland, New Zealand	3A	142.67; 192.53
Dsa, Dwa, **Dfa**	Chicago, America	5A	42.25; 64.33
Dsb, Dwb, **Dfb**	Oslo, Norway	6A	9.28; 13.06
Dsc, Dwc, **Dfc**	Anchorage, America	7	1.91; 2.54
**Dsd**, Dwd, Dfd, ET, EF	Yakutsk, Russian Federation	8	18.64; 27.61

3. CO_2 emission reduction

The CO_2 emission reduction of metric tons can be converted by electricity savings of

kilowatt-hours using the relevant factor as follows [23]:

$$1kWh = 7.09 \times 10^4\ metric\ tons\ of\ CO_2 = 0.709\ kg\ of\ CO_2$$

4. Optical parameters of smart window in the simulation project

Table 4: optical transmittance at different wavelengths

	**Whole-span average transmittance ($T_{380\text{-}1700\ nm}$)**	**Visible average transmittance ($T_{380\text{-}800\ nm}$)**	**NIR average transmittance ($T_{800\text{-}1700\ nm}$)**
**Initial**	69.1155%	62.1065%	72.3924%
**-5.0 V**	30.8704%	35.8358%	28.5434%
**-5.5 V**	7.7394%	9.8509%	6.7508%
**baseline**	87.7383%	87.0420%	87.9228%

References:

1. Granqvist CG. *Handbook of inorganic electrochromic materials*. Amsterdam: Elsevier, 1995.
2. Kresse G, Furthmüller J. Efficient iterative schemes for ab initio total-energy calculations using a plane-wave basis set. *Phys Rev B* 1996; **54**: 11169.
3. Perdew JP, Burke K, Ernzerhof M. Generalized gradient approximation made simple. *Phys Rev Lett* 1996; **77**: 3865.
4. Liechtenstein A, Anisimov VI, Zaanen J. Density-functional theory and strong interactions: Orbital ordering in Mott-Hubbard insulators. *Phys Rev B* 1995; **52**: R5467.
5. Anisimov VI, Zaanen J, Andersen OK. Band theory and Mott insulators: Hubbard U instead of Stoner I. *Phys Rev B* 1991; **44**: 943.
6. Anisimov VI, Solovyev I, Korotin M *et al.* Density-functional theory and NiO photoemission spectra. *Phys Rev B* 1993; **48**: 16929.
7. Koçer CP, Griffith KJ, Grey CP *et al.* First-principles study of localized and delocalized electronic states in crystallographic shear phases of niobium oxide. *Phys Rev B* 2019; **99**: 075151.
8. Heyd J, Scuseria GE, Ernzerhof M. Hybrid functionals based on a screened Coulomb potential. *J Chem Phys* 2003; **118**: 8207-8215.
9. Lindström H, Södergren S, Solbrand A *et al.* Li^+ ion insertion in TiO_2 (anatase). 2. Voltammetry on nanoporous films. *J Phys Chem B* 1997; **101**: 7717-7722.
10. Augustyn V, Come J, Lowe MA *et al.* High-rate electrochemical energy storage through Li^+ intercalation pseudocapacitance. *Nat Mater* 2013; **12**: 518-522.

11. Barnes P, Zuo Y, Dixon K *et al.* Electrochemically induced amorphous-to-rock-salt phase transformation in niobium oxide electrode for Li-ion batteries. *Nat Mater* 2022; **21**: 795-803.
12. Košutová T, Horák L, Pleskunov P *et al.* Thermally-driven morphogenesis of niobium nanoparticles as witnessed by in-situ x-ray scattering. *Mater Chem Phys* 2022; **277**: 125466.
13. Özer N, Barreto T, Büyüklimanli T *et al.* Characterization of sol-gel deposited niobium pentoxide films for electrochromic devices. *Sol Energy Mater Sol Cells* 1995; **36**: 433-443.
14. Nowak A, Persson J, Schmelzer B *et al.* Low temperature reduction in Ta–O and Nb–O thin films. *Phys D: Appl Phys* 2014; **47**: 135301.
15. Cui H, Zhu G, Xie Y *et al.* Black nanostructured Nb_2O_5 with improved solar absorption and enhanced photoelectrochemical water splitting. *J Mater Chem A* 2015; **3**: 11830-11837.
16. Seo J, Jeong S, Kim S. Visible-Light-Driven Water Splitting over Particulate $LaNbON_2$ Prepared from La-Rich Lanthanum Niobium Oxides. *ACS Appl. Energy Mater* 2021; **4**: 3141-3150.
17. Hojamberdiev M, Zahedi E, Nurlaela E *et al.* The cross-substitution effect of tantalum on the visible-light-driven water oxidation activity of $BaNbO_2N$ crystals grown directly by an NH_3-assisted flux method. *J. Mater Chem A* 2016; **4**: 12807-12817.
18. Darlinski A, Halbritter J. Angle‐resolved XPS studies of oxides at NbN, NbC, and Nb surfaces. *Surf Interface Anal* 1987; **10**: 223-237.
19. Shaheen BS, Hafez AM, Murali B *et al.* 10-fold enhancement in light-driven water splitting using niobium oxynitride microcone array films. *Sol Energy Mater Sol Cells* 2016; **151**: 149-153.
20. U.S. Department of Energy. Commercial Reference Buildings. https://www.energy.gov/eere/buildings/commercial-reference-buildings.
21. Bec H, McVicar T, Vergopolan N., *et al*. High-resolution (1 km) Köppen-Geiger maps for 1901–2099 based on constrained CMIP6 projections. *Sci Data* 2023; 10:724.
22. Kottek M, Grieser J, Beck C *et al.* World map of the Köppen-Geiger climate classification updated. 2006.
23 U.S. Environmental Protection Agency. Greenhouse Gas Equivalencies Calculator - Calculations and References. https://www.epa.gov/energy/greenhouse-gases-equivalencies-calculator-calculations-and-reference.
